# Monitoring Redox Pathways and Performance Limitations in Lithium‐Sulfur Batteries Using In Situ ^7/6^Li and ^33^S NMR Spectroscopies

**DOI:** 10.1002/anie.202525050

**Published:** 2026-04-22

**Authors:** Jana B. Fritzke, Sunita Dey, Christopher A. O’ Keefe, Jeongjae Lee, Kazuhiro Kamiguchi, Daisuke Mori, Yuri Nakayama, Clare P. Grey

**Affiliations:** ^1^ Yusuf Hamied Department of Chemistry University of Cambridge Cambridge UK; ^2^ The Faraday Institution Quad One Harwell Science and Innovation Campus Didcot UK; ^3^ Department of Chemistry Advanced Centre for Energy and Sustainability (ACES) University of Aberdeen Aberdeen UK; ^4^ School of Chemistry University of Hyderabad Hyderabad India; ^5^ School of Earth and Environmental Sciences Seoul National University Seoul South Korea; ^6^ Murata Manufacturing Co., Ltd. Nagaokakyo, Kyoto Japan

**Keywords:** degradation, lithium‐sulfur battery, operando NMR spectroscopy, polysulfides

## Abstract

Lithium‐sulfur (Li‐S) batteries offer high capacity and reduced costs in comparison to the traditional lithium‐ion systems. However, the complex series of redox mechanisms that occur in this battery chemistry and accompanying structural transformations are often associated with different routes for cell failure. Therefore, a fundamental understanding of the underlying mechanisms is essential to accelerate the development of these batteries. The combination of *operando* ^6/7^Li and ^33^S NMR spectroscopy is reported for the first time, providing real‐time structural information on the reaction pathways of the sulfur redox processes. The evolution of the polysulfides (poly‐S) in the electrolyte and dendrite formation on the anode was monitored with ^7^Li and ^6^Li NMR spectroscopy. Via ^33^S NMR experiments, the exact onset of Li_2_S formation was determined. By following the evolution of poly‐S species and Li_2_S, we could track the entire redox pathway and identify performance‐limiting mechanisms. The accumulation of soluble poly‐S, resulting from an incomplete poly‐S to S_8_ reduction reaction during charge, was identified as one process leading to capacity fade, while degradation via a poly‐S shuttle mechanism was negligible, at least during the first few cycles.

## Graphical abstract

Lithium‐sulfur (Li‐S) batteries contain various NMR‐active nuclear isotopes, including ^7^Li, ^6^Li and ^33^S, which are sensitive to the different chemical reactions that occur during battery cycling. Herein, we use a combination of lithium and sulfur *operando* NMR spectroscopy to provide new insights at the molecular level, fundamental understanding being essential for accelerating the development of Li‐S battery technologies.

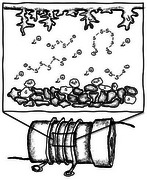

## 1 Introduction

Energy storage technologies, such as rechargeable batteries, are regarded as indispensable components of the modern energy grid, particularly when integrating renewable energy sources to balance energy supply and demand [1]. Therefore, the development of next‐generation batteries featuring higher capacity, lower costs, and improved safety is critically important. Lithium‐sulfur (Li‐S) batteries are prime candidates for next‐generation energy storage, offering a high theoretical energy density of 2600 Whkg^−1^ and benefiting from the natural abundance of sulfur [2, 3]. This has led to a widespread investigation to understand the key lithium‐sulfur redox chemistries that underpin the operation and failure modes of a Li‐S battery; this is particularly important as capacity fade during cycling is a common issue with this battery type [4].

Li‐S batteries, composed of a lithium metal anode, an elemental sulfur cathode, and an ether‐based lithium electrolyte, operate via a series of complex conversion reactions, from elemental sulfur (S_8_) to the final discharge product Li_2_S, involving an exchange of a total of 16 electrons (per S_8_ molecule) [5]. A typical voltage profile is characterized by a series of plateaus and sloping regions, which have been associated with the reduction of sulfur to form polysulfides (poly‐S) with varying chain lengths. The full reaction involves a solid‐liquid‐solid transition from solid S_8_ to dissolved poly‐S to solid Li_2_S [6, 7].

There are a number of challenges that hinder commercialization of Li‐S batteries. These include the soluble nature of the poly‐S in the electrolytes, which reduces the battery's coulombic efficiency and cycling stability due to shuttling between the cathode and anode, and the insulating nature of elemental sulfur and Li_2_S [8, 9, 10]. Degradation from the volume expansion of the cathode during electrochemical reactions leads to poor structural integrity of sulfur electrodes [11, 12, 13]. In addition, sulfur species from the cathodic reaction subsequently trigger degradation on the Li anode side. The dissolved poly‐S can chemically react with the Li metal, leading to corrosion, electrolyte consumption, deposition of Li_2_S and other solid electrolyte interphase (SEI) components (Li_2_SO_3_, Li_2_SO_4_, LiF, LiNO_x_O_y_) and non‐uniform plating [14, 15]. Thus, understanding how the many local structural changes are correlated with the (electro)chemical processes that occur during operation is crucial to improve performance, ideally by using in situ or *operando* methods.

A number of characterization methods have already been used to investigate these mechanisms, including x‐ray diffraction (XRD) [16], optical spectroscopy [17, 18], and x‐ray absorption near edge structure spectroscopy (XANES) [19]. Most of the techniques can probe only either solution‐phase or solid‐phase (amorphous or crystalline) species, but not both. Approaches capable of capturing both domains typically require specialized configurations, such as tuning the x‐ray penetration depth in XANES [20] or adjusting the laser focus in Raman spectroscopy [21]. Although depth‐resolved Raman can distinguish reaction layers in the electrolyte and electrodes, *operando* measurements necessitate an optical window and thus a dedicated cell architecture [21]. Furthermore, XANES at the sulfur K‐edge (∼2.48 keV) suffers from strong absorption, limiting its applicability to thin films or surface‐sensitive studies [20].

Nuclear magnetic resonance (NMR) spectroscopy of Li‐S batteries can offer a unique and complementary method, as it is sensitive to changes in the liquid electrolyte and to solid product formation at both the cathode and anode sides. Integrating this non‐invasive method with advanced in situ (*operando*) experimental set‐ups should enable the real‐time mapping of structural changes during (electro)chemical reactions [22]. ^7^Li NMR spectroscopy is a particularly powerful technique to apply to batteries since the chemical environments of Li‐species can be monitored during electrochemical cycling and parasitic reactions in the cell can be detected [23].

In 2014, See et al. reported the first *operando* ^7^Li NMR spectroscopy studies of the redox mechanisms of a Li‐S battery with a 1 M lithium bis(trifluoromethanesulfonyl)imide (LiTFSI) 1,3‐dioxolane (DOL), and 1,2‐dimethoxyethane (DME) electrolyte on the first discharge [24]. They tracked the formation of Li_2_S, observing that it formed almost immediately [24], likely because of the large overpotentials associated with the in situ cell. They found no evidence for any reduced sulfur‐containing solid phases other than Li_2_S, and they developed a detailed Li‐S‐electrolyte ternary phase diagram to explain their results. In 2015, Xiao et al. also reported ^7^Li NMR *operando* spectroscopy studies, their work highlighting the challenges associated with deconvoluting overlapping resonances from multiple species, in part due to the large peak broadening and shifting that arises from the bulk magnetic susceptibility (BMS) effects arising from the multiple conducting species present in the electrochemical cells [25]. This BMS effect is caused by the demagnetizing field that is induced when a sample is placed in a magnetic field (B_0_). The demagnetizing field depends on the shape of the object and its orientation with respect to B_0_, and can be particularly pronounced in heterogeneous or non‐spherical samples with large susceptibilities, such as those resulting from the paramagnetic and metallic components found in batteries [26].

The analysis and discussion of the spectra of Xiao et al. show the complexity of the various redox pathways, which include both diamagnetic and paramagnetic (radical) intermediates, their results highlighting the challenges of following these pathways by ex situ characterization [25]. Li‐containing solid species were found during their experiments, before cycling was commenced, indicating that side reactions may have already occurred [25]. Wang et al. also used *operando* ^7^Li NMR, showing good electrochemical performance of their Li‐S pouch cell, allowing them to track the formation of soluble (Li‐ion charge‐compensating poly‐S anions) and solid (Li_2_S) species over multiple cycles [4]. They outlined a four‐step pathway underlying the redox mechanism and identified the poly‐S shuttle mechanism as a main source of cell failure, solid species accumulating at the anode and cathode from the first cycle [4].

Since Li‐S batteries contain a variety of NMR‐active nuclear isotopes beyond ^7^Li—the most relevant being ^6^Li and ^33^S—tracking their changes *operando* with high time resolution should provide a comprehensive and more detailed understanding of the (electro)chemical reactions during the charge‐discharge processes in multiple cycles. ^33^S NMR spectroscopy has been largely underutilized, largely because ^33^S is an (*I* = 3/2) quadrupolar nucleus with a large quadrupole moment, a low gyromagnetic ratio, and an extremely low natural abundance of 0.76%, resulting in a very low receptivity [27]. The large ^33^S quadrupole moment results in efficient quadrupolar relaxation, particularly in liquids, leading to broad and typically undetectable NMR signals. Reasonably narrow signals can be obtained for crystalline environments where the sulfur atom is located at highly symmetric sites as found in, for example, SO_4_^2−^ or Li_2_S. Many of the difficulties encountered in the acquisition of ^33^S NMR spectra can be circumvented in part by the use of modern spectrometers operating at very high magnetic fields [28] and expensive sample enrichment.

Herein, we use for the first time a combination of lithium and sulfur *operando* NMR spectroscopy to develop a fundamental understanding of the reaction pathway of Li‐S batteries during the cycling process. In this work, we determine the exact onset potential of Li_2_S‐formation and dissolution, the latter being correlated with the appearance of soluble species in the electrolyte. In cells with excess electrolyte and Ketjen black‐based electrodes, the incomplete oxidation to elemental sulfur, including the accumulation of soluble poly‐S, leads to a significant capacity fade during cycling. These new insights at the molecular level are essential for accelerating future developments of Li‐S battery technologies.

## 2 Results

### 2.1 Electrochemical Reaction in Li‐S Batteries

**FIGURE 1:**
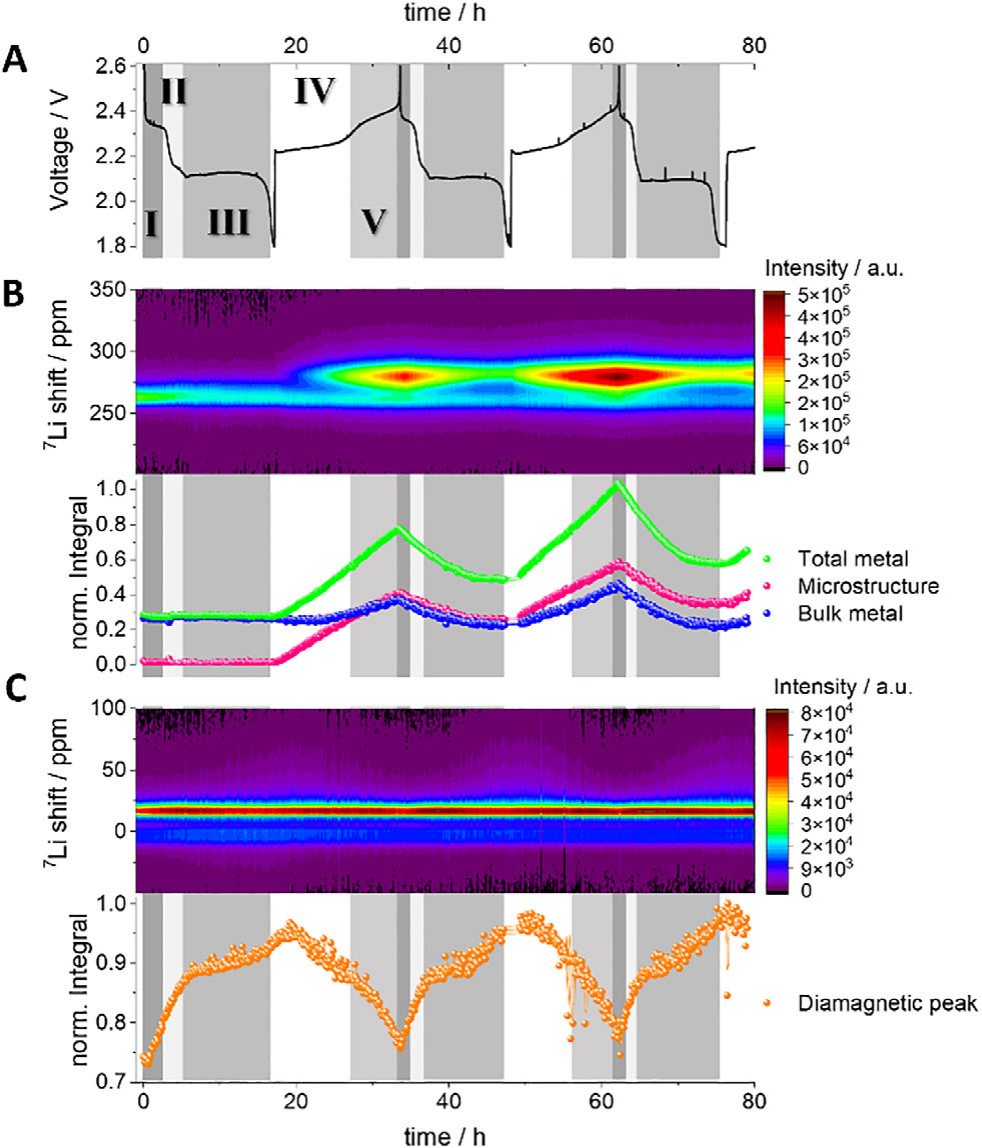
*Operando* ^7^Li NMR spectroscopy of a Li‐S battery with the corresponding voltage profile obtained when cycling with a C‐rate of C/20 shown in (A); the different voltage plateaus or processes are shaded and marked (**I–V**). (B) Plot of characteristic Li metal signals (between 200 and 350 ppm) (top) and their deconvoluted and normalized integrals (bottom). Errors (shaded region) are determined from the moving average of 5 data points. (C) Signals from diamagnetic species (between −50 and 100 ppm) (top) and their normalized integrals obtained from the moving averages of 5 data points (bottom).

A typical voltage profile and corresponding dQ/dV plot when cycling between 2.6 and 1.8 V in a 1 M LiTFSI, 0.25 M LiNO_3_ DOL:DME‐based electrolyte are shown in Figure 1A and Figure S2, respectively. The discharging process starts with a plateau around 2.33 V (Figure 1A, **I**). In this plateau, S_8_ is reduced to long‐chain poly‐S [6, 7]. These long‐chain poly‐S are successively reduced to short‐chain poly‐S during the sloping region between 2.3 V and 2.1 V, which also contains a small, flatter process just above 2.14 V (Figure 1A, **II**). The second and lower plateau around 2.13 V contributes to most of the capacity during discharge and corresponds to the reduction of poly‐S to insoluble Li_2_S [6, 7] (Figure 1A, **III**). Some cycles show a third plateau during discharge starting at 1.9 V. This third plateau was observed in previous studies using Ketjen black carbon materials and has been assigned by some authors to the slow reaction of trapped sulfur in the microporous carbon structure [29, 30]. However, the irreversible reduction of LiNO_3_ on high surface‐area cathode matrices at voltage >1.8 V cannot be excluded as a possible source of this additional discharge plateau [31].

The charging voltage profile starts with a lower plateau at around 2.23 V, corresponding to the oxidation of Li_2_S to form dissolved poly‐S on the cathode and Li metal plating on the anode (Figure 1A, **IV**). The large hysteresis in voltage between discharge and charge is largely associated with the significant overpotential/activation energy associated with the oxidation of the insulating solid phase, Li_2_S, to form poly‐S. A second plateau at around 2.38 V follows during which the different poly‐S ions are successively oxidized to elemental sulfur (Figure 1A, **V**). In addition to the electrochemical conversions, chemical reactions occur between species, often because the large overpotentials of these electrochemical reactions result in the formation of metastable, highly reactive intermediates. We now examine these processes in more detail using NMR spectroscopy.

### 2.2 ^7^Li NMR Spectroscopy

The *operando* ^7^Li NMR spectrum of a Li‐S battery shows signals in two distinct regions. The diamagnetic region around 0 ppm contains signals that are assigned to solvated Li‐ions in the electrolyte and solid components including the SEI (e.g., LiF, Li_2_O and Li_3_N) [32] on the lithium metal and Li_2_S. The metallic region at approximately 250 ppm has signals that can be assigned to the Li metal. The large shift of these species away from the diamagnetic signals is caused by the Knight shift, which arises from the interactions of the nuclear spins with the unpaired electrons at the Fermi level of the metal [23].

An analysis of the metallic region (220 to 310 ppm; labeled as “Total metal” [green]) during cycling shows a characteristic and reproducible intensity profile, with the peak broadening and increasing in intensity during charging as Li metal is plated and decreasing during the discharging processes as Li is consumed (Figure 1B). An increase during charging is attributed to the increase in surface area due to the formation of microstructured Li metal during plating (charging): the radiofrequency (rf) field from the rf pulses only penetrate the subsurface region of approximately 12 µm [33] of the Li metal in our experiment (known as the skin depth), and the total metal signal is, therefore, proportional to the surface area of the metal and not to its volume [33]. The plated Li metal, referred to as microstructure (magenta), and the bulk metal (blue) signals can be distinguished due to noticeable differences in their BMS shifts. When aligning the Li‐S cell perpendicular to the static magnetic (B_0_) field, a ^7^Li NMR resonance at around 260 ppm is seen for the pristine metal (bulk metal) [34]. During charging the cell, a new peak at around 270 ppm appears that continues to grow until the voltage reaches 2.6 V. This new resonance is characteristic of microstructured Li metal, which is less dense than the bulk metal (comprising filaments of diameter 1–2 µm), as confirmed with post‐mortem scanning electron microscopy (SEM) investigations (Figure S3). The rf field can penetrate these microstructures completely, and the detection of microstructure formation by means of NMR is, therefore, fully quantitative. During the first discharge, the intensity of the metal signal does not change. This indicates that the Li stripping process from metal in the first cycle leads to a smooth surface with very little pitting and no detectable increase in surface area (Figure 1B, bottom). The higher frequency microstructure peak appears on the onset of charging, and its intensity increases almost linearly until the end of the charging process. At the same time, the bulk metal peak increases slightly. This increase is tentatively ascribed to the formation of a dense microstructure on the surface of the strip of Li metal. The intensities of the peaks for both bulk and microstructure decrease linearly until reaching the voltage minimum of 1.8 V. After the second discharge, the microstructures are not completely consumed, and some Li remains as dendrites or dead lithium in the cell. During the second charge of the Li‐S battery, an increase of the microstructure and bulk metal peak intensity is detected again. However, microstructured Li metal is clearly seen to accumulate during the cycling process, this accumulation potentially leading to hazardous situations, such as (soft) short circuits [35, 36].

**FIGURE 2:**
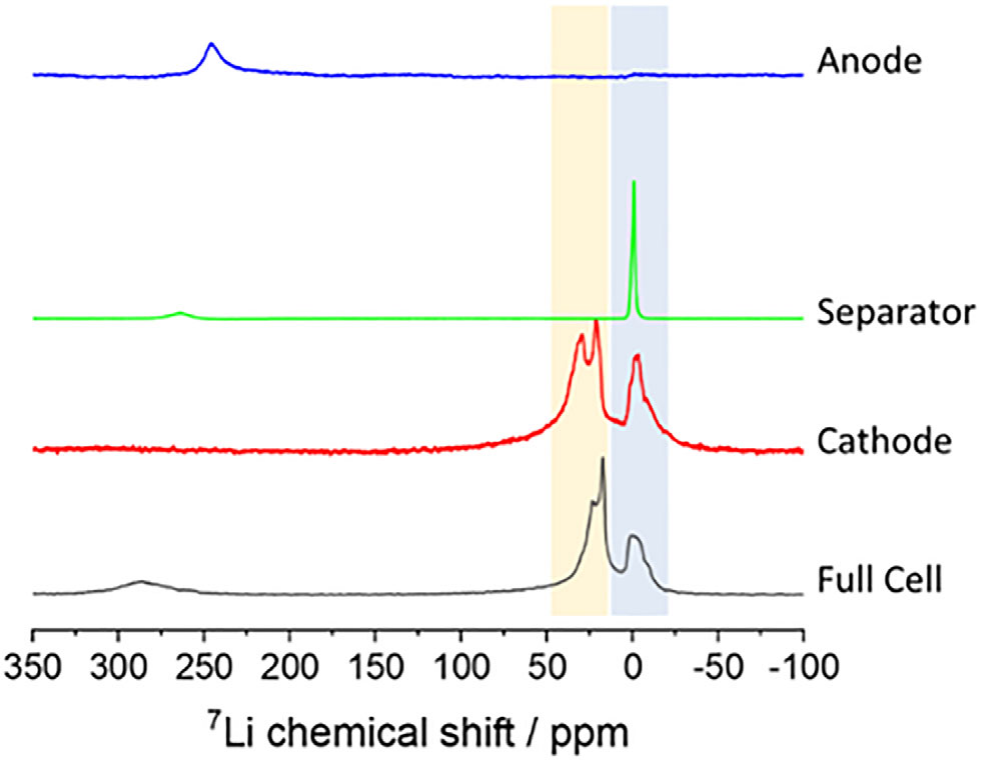
Comparison between the ^7^Li NMR spectra of an intact full cell after the third discharge with the individual cell components of a Li‐S battery after disassembly: anode (blue), separator (green), cathode (red), and intact full cell (gray) from top to bottom. The shaded diamagnetic peak regions around 20 ppm (yellow) and around ‐5 ppm (blue) highlight the different contributions of the components to the full cell spectrum. The three individual components were measured in a capsule cell at the same orientation to the magnetic field as the full cell (90°).

Multiple overlapping signals with two distinct chemical shift ranges are seen in the diamagnetic region: one range at around 20 ppm containing an intense, sharper feature, and a broader, weaker signal at approximately −5 ppm (see Figure 1C, top, and Figure 2, bottom, for the spectrum after the third discharge). A change in peak shifts and shapes is seen on rotating a fully discharged cell in the coil (Figure S5), with the sharp feature at 20 ppm shifting toward lower chemical shifts and the peaks at −5 ppm barely changing. These orientation‐dependent shifts indicate that a dominant cause of the two separate peaks close to 0 ppm is from BMS effects [26, 37] (rather than differences in chemical shifts); these BMS effects arise from variations in local magnetic fields induced by different components in the cell with very different susceptibilities (e.g., the metallic parts such as the current collector and the carbons in the cathode). As a result, the Li‐ions experience different local magnetic fields in different parts of the battery.

To obtain more information about the location of the diamagnetic species, the cell was disassembled after the third discharge, and each component of the cell was measured separately but at the same orientation as that found in the full cell (Figure 2). The spectrum of the cathode (red trace) shows a very broad diamagnetic peak, with similar features at 20 ppm (yellow) and ‐5 ppm (blue) to those observed in the spectrum of the full cell. On this basis we suggest that the carbon in the cathode is the largest source of the BMS shift of the peak at 20 ppm, carbon having a large anisotropic diamagnetic susceptibility due to its two‐dimensional atomic and electronic structure [34]. Species adsorbed on the surface or in the pores of the carbon have been reported to experience nucleus‐independent chemical shifts (NICS) to lower frequencies due to the magnetic fields induced by the ring currents [38]. The BMS shifts here are likely a result of the susceptibility of the whole carbon particles, and the different shifts may reflect Li environments within the porous electrode and outside.

The spectrum of the separator (green trace), which is soaked with the electrolyte, shows only one sharp signal at around 0 ppm. The electrolyte in the separator can also experience BMS shifts, but these will be less significant than the shift seen in carbon materials [33] and will be much smaller when the separator is physically separated from the carbon electrode. Only an extremely weak signal due to diamagnetic signals is detected in the spectrum of the anode; therefore, it can be concluded that no significant Li_2_S formation due to a poly‐S shuttle occurs on the anode surface at least after the third discharge; the SEI on Li must also be thin.

Given the difficulty in deconvoluting and assigning the signals to separate individual species in the diamagnetic region, the total intensity of all the peaks (namely, the integral of the diamagnetic region between −50 and 100 ppm) was plotted (Figure 1C, bottom). A large and steady increase of the integral of these peaks is detected in plateau **I** (2.35 V) of the first discharge and the subsequent short sloping region **II** between 2.33 and 2.11 V. This increase in integral is largely caused by an increase in intensity (and accompanying sharpening) of the peak at 20 ppm (Figure S6). This can be explained by the reduction of elemental sulfur to form soluble long‐chain poly‐S species, which dissolve into the electrolyte [39]. Since this reaction is accompanied by the oxidation of Li at the anode to form solvated Li‐ions with a variety of coordination environments depending on the state‐of‐charge (SOC), an increase in the concentration of Li‐ions in the electrolyte is observed. An increase in Li‐ions in the cathode and near the carbon will be present since these are needed for charge compensation of the negatively charged poly‐S anions. At the beginning of the 2.1 V‐discharge plateau, **III,** until the end of the discharge (1.8 V), there is a noticeable change in the rate of increase of the integral of the diamagnetic peaks, the integral increasing only slightly. The signal intensity of the 20 ppm peak decreases, and a significant broadening of the entire diamagnetic peak is detected (Figure S6). This is ascribed to the formation of both short‐chain poly‐S radicals and insoluble species from the already formed long‐chain poly‐S. The unpaired electrons in stable radicals can lead to broadening of the ^7^Li signals, either via relaxation mechanisms [40] or by electron transfer reactions between radicals and diamagnetic species [41]. In addition, solid components generally give broader signals due to the presence of anisotropic NMR interactions (these interactions are averaged out to zero or to an isotropic value in solution due to rapid molecular tumbling). The broad signals can become partially buried in the baseline, not contributing to the measurement of intensity made by integrating over the linewidth of the spectral region containing the sharper components. These solid components are, however, detected in the spectra via the periodic broadening of the diamagnetic peak that is seen to occur during the cycling (Figure 1C, top, and Figure S6). This broadening, largely coming from strong homonuclear dipolar coupling, in the case of ^7^Li, can lead to inaccurate quantification of the solid species in the diamagnetic peak (Figure S4). We, therefore, conclude that the main contribution to the intensity increase of the peak at 20 ppm is from the dissolved Li‐ions that charge‐compensate the poly‐S anions in the first half of the discharge and that these are largely located nearby the carbon as indicated by the strong BMS effect (Figure S5); the broadening and intensity decrease mainly arise from the formation of solid species, removing dissolved Li‐ions from the electrolyte in the second half of the discharge.

At the start of charging, and at the beginning of a plateau‐like region **IV** at approximately 2.2 V, the integral of the diamagnetic peaks increases sharply and continues to grow until the cell voltage reaches 2.23 V. This increase in intensity appears largely to be associated with a peak at 20 ppm and is also accompanied by a sharpening of this peak (Figure S6). We ascribe this to the oxidation of Li_2_S to form soluble poly‐S and Li‐ions in the electrolyte. Of note, the intensity is at a maximum at this point, illustrating a hysteresis in the reaction pathways. The intensity then drops steadily until approximately 2.3 V. A second plateau **V** is seen at approximately 2.38 V during the second half of the first charge and is characterized by a higher rate and nearly linear decrease of the intensity until the end of the cycle. This decrease in intensity of dissolved diamagnetic Li is due to the plating of Li metal on the anode, which is accompanied by the formation of elemental sulfur on the cathode.

The changes of the ^7^Li signal intensity during the entire cycling process are accompanied by changes of the *T_1_* (spin‐lattice) relaxation times of the spins that give rise to the two diamagnetic regions. These changes of *T_1_* are ascribed to a change of sulfur chain length, Li^+^ concentration, and radical formation (see electron paramagnetic resonance (EPR) measurements, Figure S7B), which will affect viscosity and the local fluctuating fields (seen by the ^7^Li spins) during cycling as further discussed in the Supporting Information (Figure S7). Most importantly, the *T_1_* times for the species associated with the two peaks are very different, providing further evidence that they arise from different Li local environments.

After reaching the top of charge (2.6 V), the intensity of the diamagnetic peak is 3% higher than at the beginning of the discharge. This can be explained by the presence of more Li^+^‐species in the electrolyte than present initially and/or the presence of SEI components on the anode/species resulting from a poly‐S shuttle. The intensity changes are very similar during the second cycle (as shown in Figure 1), and the total intensity of the diamagnetic peak is now 5% higher than the initial intensity, again indicating the presence of irreversible reactions.

### 2.3 ^6^Li NMR Spectroscopy

The breadth of the static ^7^Li NMR spectra is usually dominated by quadrupolar interactions and dipolar coupling, resulting in broadened line shapes. A straightforward way to improve resolution is to use ^6^Li NMR spectroscopy, because both sources of broadening are significantly reduced for spin‐1 nuclei, with their lower gyromagnetic ratio and quadrupole moment, potentially allowing overlapping signals to be resolved. Thus, a series of batteries were constructed where the natural abundance Li metal anode was replaced with a ^6^Li‐enriched metal anode, since the natural abundance of ^6^Li is only approximately 7% [27]. The electrolyte was not, however, enriched in ^6^Li. The chemical shift ranges are nominally the same between the two isotopes, and hence the ^6^Li NMR spectrum of the full battery shows the same metallic and diamagnetic peaks as observed in ^7^Li NMR spectrum [42, 43].

**FIGURE 3:**
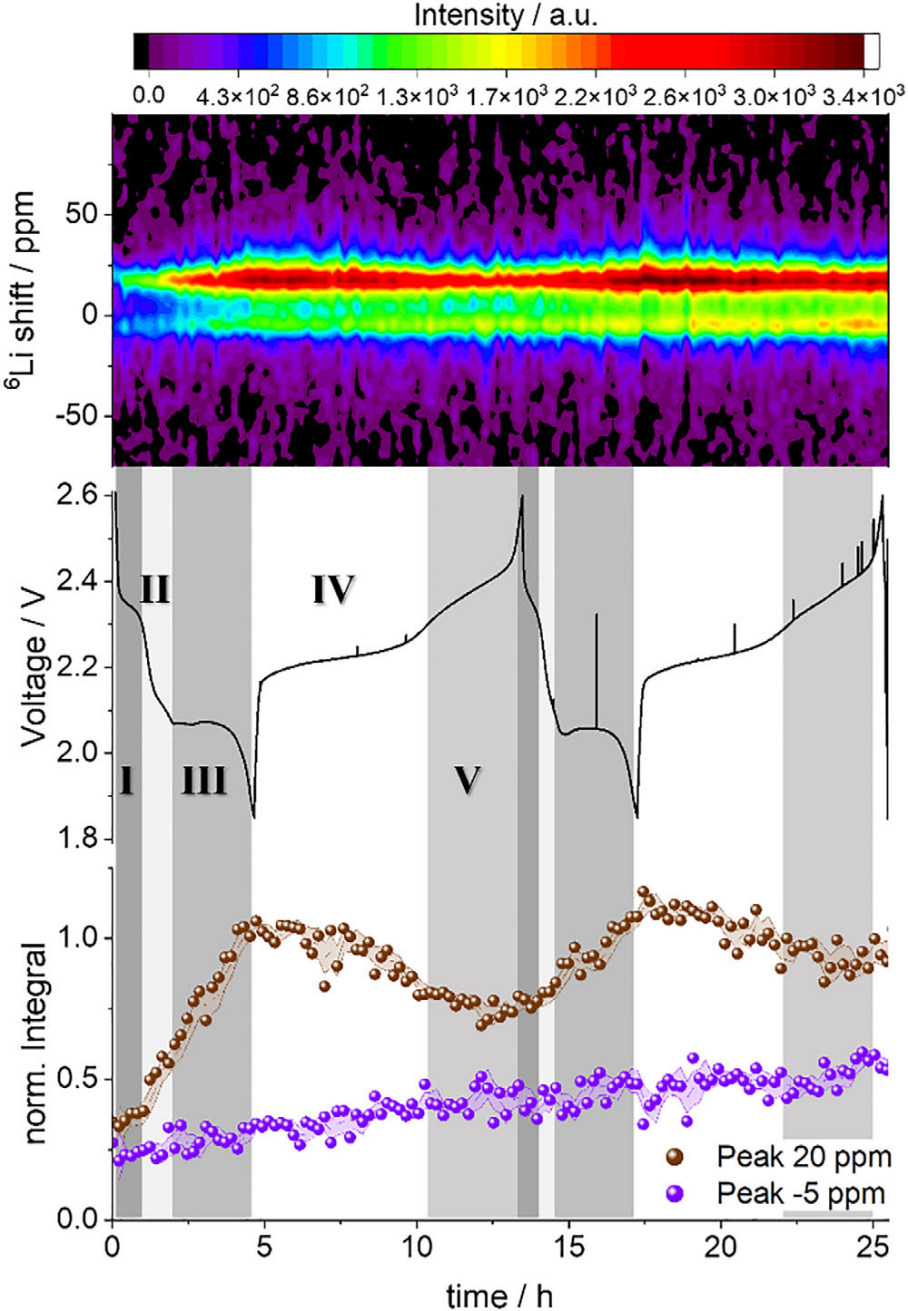
*Operando* ^6^Li NMR spectroscopy of a Li‐S battery and corresponding voltage profile with a C‐rate of C/10 with the different plateaus shown in gray (**I‐V**) (middle). The diamagnetic region (between −50 and 100 ppm) is shown on the top, and the deconvoluted and normalized integrals with the errors (shaded region) arising from the moving average of 5 data points of the peaks corresponding to diamagnetic species are plotted on the bottom.

The diamagnetic region now shows two resolved signals at around 20 ppm and at ‐5 ppm (Figure 3, top), and the evolution of the intensity of these two peaks highlights the different contribution of these two ^6^Li environments to the redox mechanism (Figure 3, bottom). The nearly linear increase in peak intensity at 20 ppm during discharge is consistent with the formation of poly‐S and the oxidation of Li^0^ at the anode, which leads to an accumulation of diamagnetic species (poly‐S and Li_2_S) in the cell until the end of the discharge, as discussed in the context of the ^7^Li NMR spectra. Surprisingly, the increase of intensity is not seen clearly until the sloping region (**II**); yet, in the voltage plateau at 2.3 V (**I**), sulfur is reduced to form long‐chain poly‐S‐ and this must be accompanied by oxidation to form dissolved Li‐ions at the anode. We ascribe these NMR observations to the rapid exchange between the 93% naturally abundant ^7^Li ions in the electrolyte and the ^6^Li metal, which starts immediately after cell assembly [33]. A rate for this exchange of around 10^−6^ mol m^−2^ s^−1^ has been estimated [33], and as a result of this exchange, the surface of the ^6^Li metal anode is almost immediately enriched with ^7^Li. This hypothesis was confirmed by an experiment where a ^6^Li metal anode was used, but the ^7^Li nuclei were detected (Figure S8). Here a rapid decrease in the ^7^Li metal signal was seen as these surface ^7^Li atoms are oxidized, which was accompanied by a small increase in the intensity of the diamagnetic peaks during the 2.3 V discharge plateau (**I**). A steady decrease of the diamagnetic peak integral was observed as the ^6^Li/^7^Li exchange processes continue to occur, but more bulk ^6^Li metal atoms are oxidized.

A slight broadening of the peak at 20 ppm was detected during discharge, which can be explained by the formation of Li_2_S. However, the detection of Li_2_S is again not likely to be fully quantitative during the *operando* experiment, because the relaxation times of most ^6^Li solids are very long, often up to hundreds of seconds [44]. Thus, we ascribe the major changes to the intensity of the peak at 20 ppm during discharge to the formation of the soluble ^6^Li^+^‐species, especially during the second discharge plateau, the formation of soluble poly‐S at the beginning of the discharge being obscured by the ^7^Li^+^ electrolyte/ ^6^Li metal exchange reaction.

During the first half of the charging plateau (**IV**), the intensity and integral of the peak at 20 ppm stay constant, indicating that the consumption rate of diamagnetic ^6^Li‐ions due to metal plating is nearly equal to the formation of soluble diamagnetic ^6^Li‐ions due to Li_2_S oxidation. From the second half of plateau **IV** until the end of the charging process, the integral decreases, which is explained by the continuous plating of Li metal from diamagnetic species, while elemental sulfur is formed. In contrast to the peak at 20 ppm, the peak at −5 ppm shows a monotonic increase of the integral during cycling. That suggests that this signal originates from species not directly involved in the different redox mechanisms. The gradual increase is ascribed to the accumulation of ^6^Li^+^ (originally in the ^6^Li metal anode) in the electrolyte with Li^0^ stripping and plating and the exchange with the ^7^Li‐ions originally in the dissolved electrolyte salts (LiTFSI and LiNO_3_). There is also an accumulation of residual poly‐S in the electrolyte. This observation supports the assignment of the diamagnetic signal regions based on their distinct BMS shifts, the signal at 20 ppm being associated with the redox active species, while the –5 ppm peak corresponds to electrolyte ions located farther from the carbon surface (largely within the separator).

### 2.4 ^33^S NMR Spectroscopy

**FIGURE 4:**
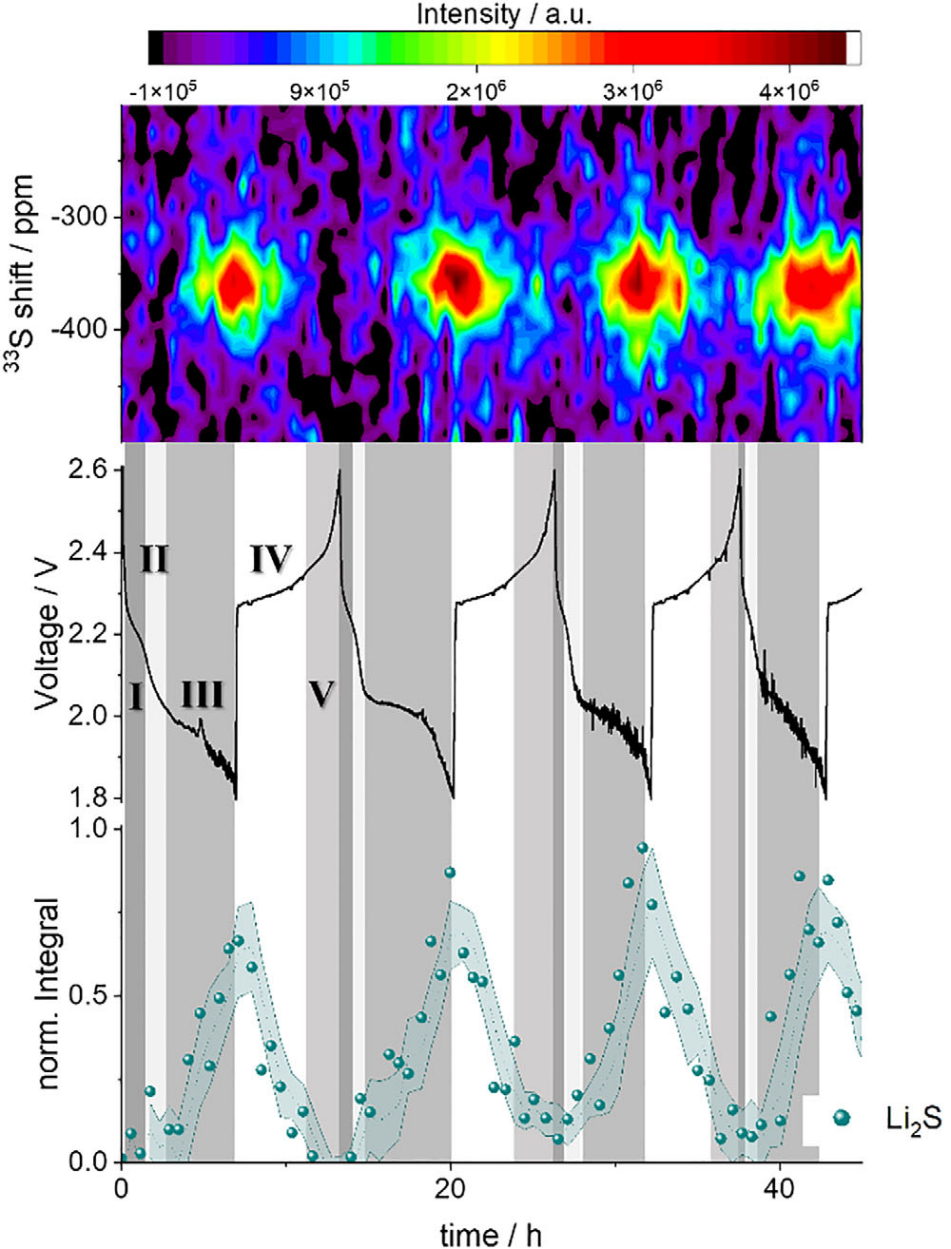
In situ ^33^S NMR spectroscopic investigation of a Li‐S battery. Signal (between −500 and −200 ppm) on the top, voltage profile with a C‐rate of C/10 with the different plateaus in gray (**I–V**) (middle), and normalized integral with the errors (shaded region) from the moving average of 5 data points of the Li_2_S peak (bottom).

On cycling a Li‐S battery with a ^33^S enriched cathode (10 wt% sulfur enriched to 99% was used—see Supporting Information for more details), a signal with a chemical shift centered at ‐355 ppm, characteristic of Li_2_S [45], is seen, which appears and disappears during cycling (Figure 4, top). No signals from poly‐S environments are seen, presumably because the significant relaxation induced by the large quadrupolar coupling constants of these ^33^S environments broadens the signals so that they are not detectable. The quantification of the Li_2_S signal shows a characteristic integral profile (Figure 4, bottom) with Li_2_S formation starting from the beginning of the 2.1 V‐discharge plateau (**III**), the integral increasing linearly until the end of the discharge. From the start of the charging process, the Li_2_S peak integral decreases nearly linearly until it is completely consumed in the first charging plateau (**V**). The *operando* ^33^S NMR shows that the formation and consumption of Li_2_S in the battery is almost completely reversible (within the signal‐to‐noise S/N) in the following cycles, indicating that our cell does not suffer from accumulation of inactive Li_2_S inside of the cell in the first two cycles. Furthermore, ^33^S NMR experiments of the cell components obtained from a disassembled cell detected Li_2_S in the cathode (and separator) with no accumulation of Li_2_S on the anode (arising from a poly‐S shuttle; Figure S9) after the second discharge. Analysis of subsequent cycles reveals a small but measurable accumulation of Li_2_S in the cell (see Figure S10). However, the detected amount of Li_2_S is significantly lower than expected from the observed capacity fade, indicating that Li_2_S accumulation is not the dominant degradation mechanism during the initial stages of cycling.

## 3 Discussion

**FIGURE 5:**
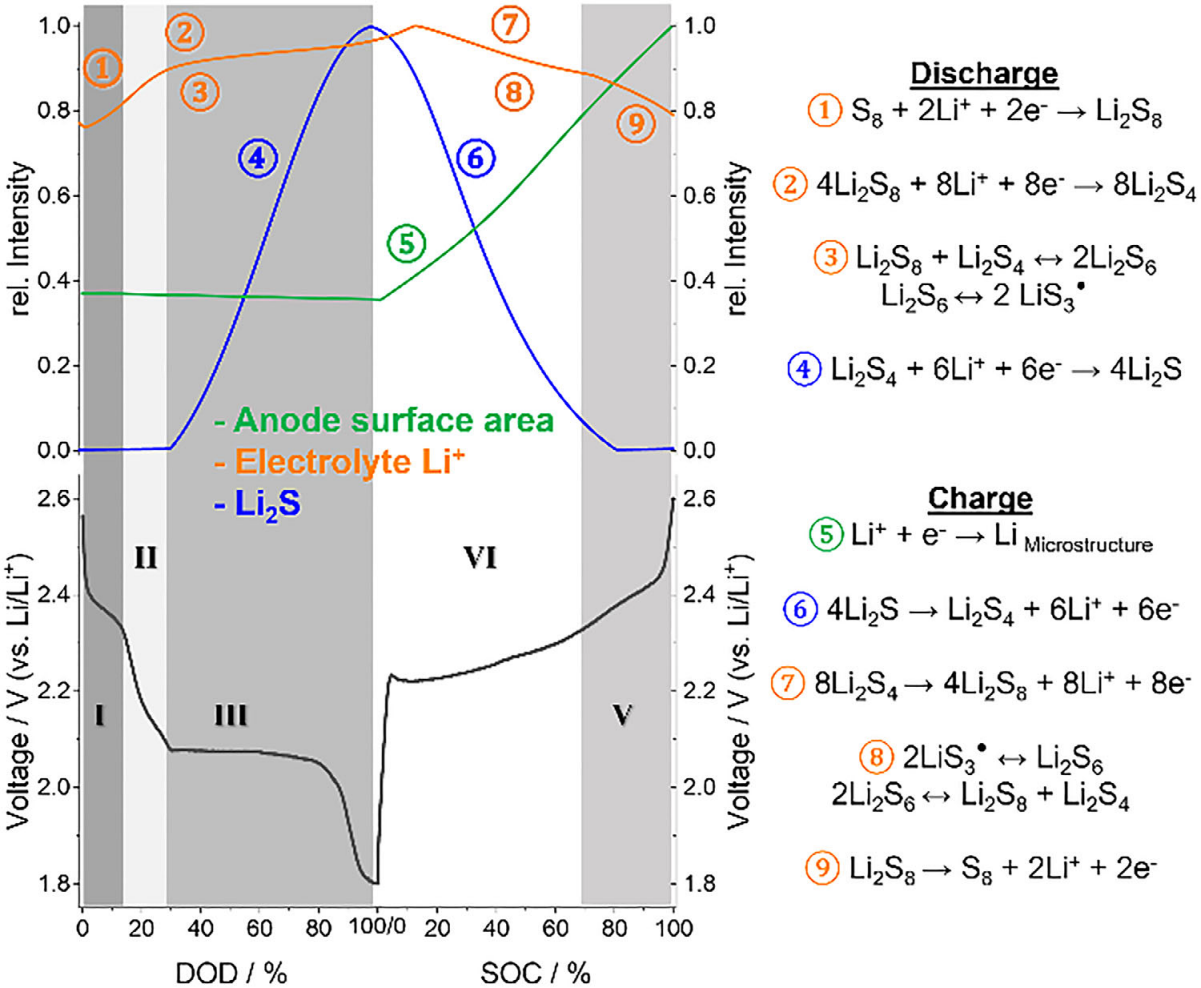
The characteristic voltage profile (dark gray, bottom) during the first cycle of a Li‐S battery, and, at the top, the change in NMR intensities of the signals corresponding to the observed species. Different reaction products were identified during the discharging and charging process with the help of the NMR experiments, and reactions are given on the right‐hand side. The diamagnetic ^7^Li peaks between −50 and 100 ppm (orange) correspond to the successive reduction reactions of S_8_ to form different dissolved polyanion species. The formation and dissolution of Li_2_S is tracked via the ^33^S NMR (blue). The formation of higher surface area Li metal is shown in green (Li^0^ resonance).

O*perando* NMR spectroscopic measurements of the NMR‐active nuclei of the redox‐active species, Li and S, allow the reactions and the transient species inside the Li‐S cells to be followed. Furthermore, the species that accumulate during cycling and that may be responsible for capacity degradation in Li‐S batteries can be tracked with high time resolution. Figure 5 summarizes the changes in intensity of the various ^7^Li and ^33^S signals with state‐of‐charge (SOC) and depth‐of‐discharge (DOD), and we now discuss the mechanistic conclusions that we can draw from these results.

Our ^7^Li NMR spectroscopy experiments show that the formation of soluble redox‐active species starts from the beginning of the 2.3 V‐discharge plateau (**I**), as seen via a rapid and steady increase in the integral of the diamagnetic ^7^Li NMR peaks assigned to dissolved Li‐ions during this plateau. These Li‐ions charge‐compensate the formation of negatively charged long‐chain poly‐S (e.g., Li_2_S_8_ and Li_2_S_6_) that are formed from the ring‐breaking of S_8_ (Figure 5, orange ①) [46].

Since assigning and deconvoluting the signals in the diamagnetic region is not straightforward with ^7^Li NMR spectroscopy, ^6^Li NMR was also used to monitor the formation of Li‐ions that accompany the formation of soluble long‐ and short‐chain poly‐S charge. These are associated with the peak at 20 ppm in the diamagnetic region, which is assigned to dissolved electrolyte species nearby the carbon electrode, along with a component from solid Li_2_S. The increase in ^6^Li signal intensity that should accompany the formation of long‐chain poly‐S is hidden at the beginning of the first discharge by the exchange between ^7^Li ‐ions in the electrolyte and ^6^Li^0^ in the Li metal, since we did not use a ^6^Li‐enriched electrolyte. As a result, the first ions to be stripped from the Li metal are largely ^7^Li‐ions. An increase in the intensity of the 20 ppm peak is, however, detected starting from sloping region **II** until the end of the discharge, confirming that this signal is associated with a redox (charge compensation) mechanism. The second diamagnetic region at the lower frequency (–5 ppm) is assigned to Li^+^‐species that do not directly contribute to the redox process, such as dissolved Li‐ions (largely in the separator) or diamagnetic SEI components clearly shown in the ^6^Li experiment.

Focusing on the 20 ppm peak in the ^7^Li spectra, it shows an intensity increase (and peak sharpening) during plateau **I** and the sloping region **II** (Figure S6), due to the formation of long and short chain poly‐S, the latter being preferred stabilized in this DOL:DME‐based electrolyte system [17]. The long‐chain poly‐S undergoes electrochemical reduction to form short‐chain poly‐S (e.g. Li_2_S_4_) in region **II** (Figure 5, orange ②) [6]. In addition to this electrochemical decomposition, the long‐chain poly‐S undergo a rapid comproportionation reaction between the formed Li_2_S_4_ and the remaining Li_2_S_8_ yielding Li_2_S_6_ (Figure 5, orange ③), previously reported by UV/Vis and EPR studies [6, 47]. All of these reactions involve the formation of more dissolved Li‐ions (one Li ion being formed per electron), which we can directly monitor in our NMR experiments. Li_2_S_6_ has been reported (on the basis of a previous *operando* EPR study) to break down to form LiS_3_^•^ during the sloping region; we also observed a drop in the ^7^Li *T_1_* times in the sloping region at 28% DOD (Figure S7), which we tentatively ascribe to the formation of LiS_3_^•^ [47].

Given the difficulty of differentiating between the different sulfur species with ^6,7^Li NMR, we used ^33^S NMR spectroscopy to follow the formation and consumption of Li_2_S, which is the insoluble endpoint of the S‐redox process. The electrochemical formation of Li_2_S from Li_2_S_4_ and other dissolved species starts at the beginning of the 2.1 V‐discharge plateau (**III**) at around 30% DOD (Figure 5, blue ④), as seen in the ^33^S spectra. At the same time, the ^7^Li diamagnetic signal increases in intensity, but at a slower rate. The formation of solid Li_2_S is not quantitative in the ^6,7^Li spectra under the conditions used here, because of the broad peaks associated with this solid product in addition to its likely longer *T_1_*. From 30% DOD until 100% DOD all reactions ①–④ occur at the same time, and soluble and insoluble poly‐S species coexist in the cell, consistent with the steady increase of the intensities of the signals from the diamagnetic ^7^Li‐ions and the ^33^S signal from Li_2_S.

We note that the total capacity of process **I** corresponds to only approximately 15% of the theoretical capacity of the Li‐S battery shown in Figure 2 (cycled at C/20) corresponding to a stoichiometry of the sulfur species of Li_0.3_S_2_ (i.e., 0.6 Li_2_S_8_ + 0.4 S_8_ with 100% DOD corresponding to Li_2_S), assuming 100% coulombic efficiency. At this C‐rate, the dissolved polyanions either diffuse far enough from the carbon electrode and/or a hindered diffusion through the porous cathode structure slows down the redox reactions so that a complete reaction to form Li_2_S_8_ is not achieved in process **I**. The dissolved species do not diffuse far, however, because it is the 20 ppm peak that undergoes the largest change in intensity, not the peak at close to −5 ppm (largely from ions in the separator), consistent with the very little, if any, movement of poly‐S into the separator. During process **II**, reactions to form the shorter chain polysulfides such as Li_2_S_6_ and Li_2_S_4_ start to occur as the voltage drops steadily, but the oxidation of the residual S_8_ must also continue to occur concomitantly, and the stoichiometry of the sulfur species at the end of process **II** is now Li_0.6_S_2_ (i.e., Li_2_S_8_ + 0.1 Li_2_S_4_ assuming complete S reaction). At C/10 cycling (Figure 3), even fewer polyanions are formed in processes **I** and **II** before the onset of Li_2_S formation.

The charging process begins with an overpotential peak in the voltage profile, which is ascribed to the nucleation of Li metal on the surface of the anode and oxidation of Li_2_S in the cathode. Li plating can be detected via the linear intensity change of the ^7^Li peak assigned to the Li^0^ microstructures until 100% SOC, plating leading to an increase of the surface area of the anode (Figure 5, green ⑤). This peak shows a higher shift than the bulk metal (due to BMS effects [23]). Li_2_S also starts to dissolve from the beginning of the charge, as seen in the ^33^S NMR (Figure 5, blue ⑥). The oxidation of Li_2_S to form Li_2_S_4_ should lead to the generation of 1 solvated Li^+^‐ion, per oxidation of two formula units of Li_2_S (involving three electrons), as seen in reaction ⑥, consistent with the increase in the electrolyte ^7^Li^+^ intensity until approximately 15% SOC. However, thereafter, the Li^+^ electrolyte intensity starts to decrease nearly linearly. In region **VI**, S_8_ has not started to form (the voltage is too low [48]), and we ascribe this decrease to the oxidation of short‐chain poly‐S, the oxidation of Li_2_S_4_ to form Li_2_S_8_ (in reaction ⑦), for example, resulting in the removal of one Li^+^ from the electrolyte per electron. Thus, if ⑥ and ⑦ occur simultaneously, a slow decrease in ^7^Li intensity is predicted, as observed experimentally. This formation of poly‐S with different chain lengths from short (Li_2_S_4_) to long (Li_2_S_8_) (Figure 5, orange ⑦–⑨) is reflected in the change of *T_1_* relaxation times, the T_1_ relaxation times of the diamagnetic Li species being influenced by the poly‐S chain lengths and viscosity of the electrolyte (Figure S7). As more Li_2_S_8_ is formed and more Li_2_S is consumed, the voltage drifts up, and in region **V**, it is sufficiently high that S_8_ may now be formed. At this point the Li_2_S is completely consumed (80% SOC). The steep decrease of the detected diamagnetic Li species in region (**V**), is consistent with the removal of dissolved poly‐S to form S_8_.

The poly‐S are not, however, completely consumed, as indicated by the gradual increase of the integral of the diamagnetic peaks in the ^7^Li and the ^6^Li NMR experiments while cycling. This poly‐S accumulation can be explained by a blocking of the electrochemically active sites (on the electrically connected carbon matrix) in the cathode by a covering of insulating sulfur and provides one explanation for the capacity fade of the battery. In addition to the sulfur redox mechanisms, the formation of microstructures on the surface of the anode and/or dead Li metal starting from the first charge process can also contribute to the performance limitation of the Li‐S battery. Li_2_S formation/consumption is not a performance‐limiting mechanism in our described cell system because very little accumulation of Li_2_S and poly‐S shuttle side products was detected.

## 4 Conclusion

In conclusion, the combination of complementary in situ NMR spectroscopic measurements of the redox‐active nuclei ^7^Li, ^6^Li, and ^33^S has been used in experiments presented in this work to provide a clear picture about the S‐redox process in the Li‐S cell and elucidate and describe the various competing mechanisms (Figure 5). This understanding of the redox process is possible because we can detect the liquid and solid species at the same time. Our work, therefore, builds on previous studies where the results from several different techniques, such as XRD, UV/Vis, Raman, and EPR spectroscopy, had to be combined to obtain a full understanding of the sulfur redox chemistry, and is a strength of our methodology. Building on previous *operando* studies [6, 47, 48], we show that even though resolution of different species in solution is challenging, by careful monitoring of the intensity of the electrolyte signals, the balance between the formation of solution and solid‐state species can be determined. Furthermore, via the BMS effects, which usually complicate interpretation of diamagnetic signals, we were able to separate the signals from solvated Li‐ions primarily in the electrolyte within the separator and those near the carbon of the cathode. Specifically, the results indicate that most of the redox‐active species remain near the carbon, since it is the ^6^Li peak at 20 ppm that grows steadily in intensity as Li‐ions generated at the anode migrate toward the cathode to charge balance the dissolved poly‐S. This, in combination with ex situ measurements of the cell components, where Li_2_S was only found on the cathode, shows that our cell systems do not suffer from poly‐S shuttle at least in the first few cycles.

*Operando* ^33^S NMR spectroscopy was used for the first time in Li‐S batteries, and the applicability of the method to detect species with small C_Q_s was demonstrated. The formation and consumption of Li_2_S were tracked via the ^33^S signal, clearly showing the reversibility of the S‐redox reaction. However, our *operando* NMR measurements showed that the consumption of poly‐S is not complete during cycling, the accumulation of poly‐S in conjunction with the formation of dendrites during the first charge and subsequent charges contributing to the observed capacity fade in our cell system. Together, the combination of ^6,7^Li and ^33^S NMR spectroscopy allows more details of the S‐redox mechanisms to be elucidated and helps identify performance‐limiting steps, both of which are essential for accelerating the development of Li‐S battery technologies.

## Conflicts of Interest

The authors declare no conflicts of interest.

## Supporting information



The authors have cited additional references within the Supporting Information [6, 7, 19, 23, 33, 34, 38, 47, 49, 50, 51, 52, 53, 54, 55].

## Data Availability

The data that support the findings of this study are available from the corresponding author upon reasonable request.

## References

[1] D. Larcher and J.‐M. Tarascon , “Towards Greener and More Sustainable Batteries for Electrical Energy Storage,” Nature Chemistry 7 (2015): 19–29, 10.1038/nchem.2085. 25515886

[2] A. Manthiram , Y. Fu , S.‐H. Chung , C. Zu , and Y.‐S. Su , “Rechargeable Lithium–Sulfur Batteries,” Chemical Reviews 114 (2014): 11751–11787. 25026475 10.1021/cr500062v

[3] Y. Yin , S. Xin , Y. Guo , and L. Wan , “Lithium–Sulfur Batteries: Electrochemistry, Materials, and Prospects,” Angewandte Chemie International Edition 52 (2013): 13186–13200, 10.1002/anie.201304762. 24243546

[4] H. Wang , N. Sa , M. He , et al., “In Situ NMR Observation of the Temporal Speciation of Lithium Sulfur Batteries During Electrochemical Cycling,” Journal of Physical Chemistry C 121 (2017): 6011–6017, 10.1021/acs.jpcc.7b01922.

[5] P. G. Bruce , S. A. Freunberger , L. J. Hardwick , and J.‐M. Tarascon , “Li–O_2_ and Li–S Batteries With High Energy Storage,” Nature Materials 11 (2012): 19–29, 10.1038/nmat3191. 22169914

[6] Y. Luo , Z. Fang , S. Duan , et al., “Direct Monitoring of Li_2_S_2_ Evolution and Its Influence on the Reversible Capacities of Lithium‐Sulfur Batteries,” Angewandte Chemie International Edition 62 (2023): e202215802. 36650422 10.1002/anie.202215802

[7] C. Barchasz , F. Molton , C. Duboc , J. C. Leprêtre , S. Patoux , and F. Alloin , “Lithium/Sulfur Cell Discharge Mechanism: An Original Approach for Intermediate Species Identification,” Analytical Chemistry 84 (2012): 3973–3980, 10.1021/ac2032244. 22482872

[8] E. Peled , I. Shekhtman , T. Mukra , M. Goor , I. Belenkaya , and D. Golodnitsky , “Improving the Durability and Minimizing the Polysulfide Shuttle in the Li/S Battery,” Journal of the Electrochemical Society 165 (2018): A6051–A6057, 10.1149/2.0101801jes.

[9] N. Kang , Y. Lin , L. Yang , et al., “Cathode Porosity Is a Missing Key Parameter to Optimize Lithium‐Sulfur Battery Energy Density,” Nature Communications 10 (2019): 4597, 10.1038/s41467-019-12542-6. PMC6787095 31601812

[10] L. Qie and A. Manthiram , “High‐Energy‐Density Lithium–Sulfur Batteries Based on Blade‐Cast Pure Sulfur Electrodes,” ACS Energy Letters 1 (2016): 46–51, 10.1021/acsenergylett.6b00033.

[11] J. Zhang , W. Ma , Z. Feng , et al., “P‐Doped BN Nanosheets Decorated Graphene as the Functional Interlayer for Li–S Batteries,” Journal of Energy Chemistry 39 (2019): 54–60, 10.1016/j.jechem.2019.01.016.

[12] Q. Qi , X. Lv , W. Lv , and Q.‐H. Yang , “Multifunctional Binder Designs for Lithium‐Sulfur Batteries,” Journal of Energy Chemistry 39 (2019): 88–100, 10.1016/j.jechem.2019.02.001.

[13] F. Zhao , J. Xue , W. Shao , H. Yu , W. Huang , and J. Xiao , “Toward High‐Sulfur‐Content, High‐performance Lithium‐Sulfur Batteries: Review of Materials and Technologies,” Journal of Energy Chemistry 80 (2023): 625–657, 10.1016/j.jechem.2023.02.009.

[14] H. Ye and Y. Li , “Room‐Temperature Metal–sulfur Batteries: What Can We Learn From Lithium–Sulfur?” InfoMat 4 (2022): e12291, 10.1002/inf2.12291.

[15] L. P. Hou , L. Y. Yao , C. X. Bi , et al., “High‐valence Sulfur‐Containing Species in Solid Electrolyte Interphase Stabilizes Lithium Metal Anodes in Lithium–Sulfur Batteries,” Journal of Energy Chemistry 68 (2022): 300–305, 10.1016/j.jechem.2021.12.024.

[16] M. Cuisinier , P. E. Cabelguen , S. Evers , et al., “Sulfur Speciation in Li–S Batteries Determined by Operando X‐Ray Absorption Spectroscopy,” Journal of Physical Chemistry Letters 4 (2013): 3227–3232, 10.1021/jz401763d.

[17] Q. Zou and Y. C. Lu , “Solvent‐Dictated Lithium Sulfur Redox Reactions: An Operando UV–vis Spectroscopic Study,” Journal of Physical Chemistry Letters 7 (2016): 1518–1525, 10.1021/acs.jpclett.6b00228. 27050386

[18] S. S. Zhang , “A New Finding on the Role of LiNO_3_ in Lithium‐Sulfur Battery,” Journal of Power Sources 322 (2016): 99–105, 10.1016/j.jpowsour.2016.05.009.

[19] M. U. M. Patel , I. Arčon , G. Aquilanti , L. Stievano , G. Mali , and R. Dominko , “X‐Ray Absorption Near‐Edge Structure and Nuclear Magnetic Resonance Study of the Lithium–Sulfur Battery and Its Components,” ChemPhysChem 15 (2014): 894–904, 10.1002/cphc.201300972. 24497200

[20] K. Miao , S. Chen , and J. Zhou , “The X‐Ray Absorption Spectroscopy for Advanced Battery Systems,” Sustainable Materials and Technologies 45 (2025): e01608, 10.1016/j.susmat.2025.e01608.

[21] L. Xue , Y. Li , A. Hu , et al., “Situ/Operando Raman Techniques in Lithium–Sulfur Batteries,” Small Structures 3 (2022), 10.1002/sstr.202100170.

[22] J. B. Richter , C. Eßbach , I. Senkovska , S. Kaskel , and E. Brunner , “Quantitative In Situ ^13^C NMR Studies of the Electro‐Catalytic Oxidation of Ethanol,” Chemical Communications 55 (2019): 6042–6045, 10.1039/C9CC02660F. 31065638

[23] R. Bhattacharyya , B. Key , H. Chen , A. S. Best , A. F. Hollenkamp , and C. P. Grey , “In Situ NMR Observation of the Formation of Metallic Lithium Microstructures in Lithium Batteries,” Nature Materials 9 (2010): 504–510, 10.1038/nmat2764. 20473288

[24] K. A. See , M. Leskes , J. M. Griffin , et al., “Ab Initio Structure Search and In Situ ^7^Li NMR Studies of Discharge Products in the Li–S Battery System,” Journal of the American Chemical Society 136 (2014): 16368–16377, 10.1021/ja508982p. 25384082 PMC4353022

[25] J. Xiao , J. Z. Hu , H. Chen , et al., “Following the Transient Reactions in Lithium–Sulfur Batteries Using an In Situ Nuclear Magnetic Resonance Technique,” Nano Letters 15 (2015): 3309–3316, 10.1021/acs.nanolett.5b00521. 25785550

[26] L. Zhou , M. Leskes , A. J. Ilott , N. M. Trease , and C. P. Grey , “Paramagnetic Electrodes and Bulk Magnetic Susceptibility Effects in the In Situ NMR Studies of Batteries: Application to Li_1.08_Mn_1.92_O_4_ Spinels,” Journal of Magnetic Resonance 234 (2013): 44–57, 10.1016/j.jmr.2013.05.011. 23838525

[27] K. J. D. MacKenzie and M. E. Smith , Multinuclear Solid‐State NMR of Inorganic Materials (Elsevier, 2002).

[28] R. Musio , “Applications of ^33^S NMR Spectroscopy,” Annual Reports on NMR Spectroscopy 68 (2009), 1–88, 10.1016/S0066-4103(09)06801-X.

[29] L. Li , Z. Ma , and Y. Li , “Accurate Determination of Optimal Sulfur Content in Mesoporous Carbon Hosts for High‐Capacity Stable Lithium‐Sulfur Batteries,” Carbon 197 (2022): 200–208.

[30] C. Yeo and M. Kim , “Enhancing Cycle Stability via Discharge Voltage Regulation in Li–S Batteries: Impact of the Lower Cutoff Voltage on Electrochemical Stability,” Journal of the Electrochemical Society 171 (2024): 060511.

[31] A. Rosenman , R. Elazari , G. Salitra , E. Markevich , D. Aurbach , and A. Garsuch , “The Effect of Interactions and Reduction Products of LiNO_3_, the Anti‐Shuttle Agent, in Li‐S Battery Systems,” Journal of the Electrochemical Society 162 (2015): A470–A473, 10.1149/2.0861503jes.

[32] R. May , K. J. Fritzsching , D. Livitz , S. R. Denny , and L. E. Marbella , “Rapid Interfacial Exchange of Li Ions Dictates High Coulombic Efficiency in Li Metal Anodes,” ACS Energy Letters 6 (2021): 1162–1169, 10.1021/acsenergylett.1c00112.

[33] A. B. Gunnarsdóttir , S. Vema , S. Menkin , L. E. Marbella , and C. P. Grey , “Investigating the Effect of a Fluoroethylene Carbonate Additive on Lithium Deposition and the Solid Electrolyte Interphase in Lithium Metal Batteries Using In Situ NMR Spectroscopy,” Journal of Materials Chemistry A 8 (2020): 14975–14992, 10.1039/D0TA05652A.

[34] N. M. Trease , L. Zhou , H. J. Chang , B. Y. Zhu , and C. P. Grey , “In Situ NMR of Lithium Ion Batteries: Bulk Susceptibility Effects and Practical Considerations,” Solid State Nuclear Magnetic Resonance 42 (2012): 62–70, 10.1016/j.ssnmr.2012.01.004. 22381594

[35] L. Shi , C. S. Anderson , L. Mishra , et al., “Early Failure of Lithium–Sulfur Batteries at Practical Conditions: Crosstalk Between Sulfur Cathode and Lithium Anode,” Advanced Science 9 (2022): 2201640, 10.1002/advs.202201640. 35524632 PMC9313511

[36] S. Menkin , J. B. Fritzke , R. Larner , et al., “Insights Into Soft Short Circuit‐Based Degradation of Lithium Metal Batteries,” Faraday Discussions 248 (2024): 277–297, 10.1039/D3FD00101F. 37870402 PMC10823489

[37] R. Pigliapochi , L. O'Brien , A. J. Pell , et al., “When Do Anisotropic Magnetic Susceptibilities Lead to Large NMR Shifts? Exploring Particle Shape Effects in the Battery Electrode Material LiFePO_4_ ,” Journal of the American Chemical Society 141 (2019): 13089–13100. 31271033 10.1021/jacs.9b04674

[38] R. K. Harris , T. V. Thompson , P. R. Norman , and C. Pottage , “Phosphorus‐31 NMR Studies of Adsorption Onto Activated Carbon,” Carbon 37 (1999): 1425–1430.

[39] S. Lang , S.‐H. Yu , X. Feng , M. R. Krumov , and H. D. Abruña , “Understanding the Lithium–Sulfur Battery Redox Reactions via Operando Confocal Raman Microscopy,” Nature Communications 13 (2022): 4811. 10.1038/s41467-022-32139-w PMC9381601 35973986

[40] M. H. Levitt , Spin Dynamics: Basics of Nuclear Magnetic Resonance (Wiley, 2008).

[41] E. W. Zhao , T. Liu , E. Jónsson , et al., “In Situ NMR Metrology Reveals Reaction Mechanisms in Redox Flow Batteries,” Nature 579 (2020): 224–228, 10.1038/s41586-020-2081-7. 32123353

[42] S. Patai and Z. Rappoport , The Chemistry of Organolithium Compounds Part 1 (John Wiley & Sons, 2004).

[43] H. J. Chang , N. M. Trease , A. J. Ilott , et al., “Investigating Li Microstructure Formation on Li Anodes for Lithium Batteries by In Situ ^6^Li/^7^Li NMR and SEM,” Journal of Physical Chemistry C 119 (2015): 16443–16451, 10.1021/acs.jpcc.5b03396.

[44] A. Gordji‐Nejad , J. Colell , S. Glöggler , B. Blümich , and S. Appelt , “Studies of ^6^Li‐NMR Properties in Different Salt Solutions in Low Magnetic Fields,” Journal of Magnetic Resonance 214 (2012): 10–14, 10.1016/j.jmr.2011.09.040. 22055979

[45] T. A. Wagler , W. A. Daunch , P. L. Rinaldi , and A. R. Palmer , “Solid State ^33^S NMR of Inorganic Sulfides,” Journal of Magnetic Resonance 161 (2003): 191–197, 10.1016/S1090-7807(03)00046-6. 12713969

[46] R. Liu , Z. Wei , L. Peng , et al., “Establishing Reaction Networks in the 16‐Electron Sulfur Reduction Reaction,” Nature 626 (2024): 98–104, 10.1038/s41586-023-06918-4. 38297176

[47] Q. Wang , J. Zheng , E. Walter , et al., “Direct Observation of Sulfur Radicals as Reaction Media in Lithium Sulfur Batteries,” Journal of the Electrochemical Society 162 (2015): A474–A478, 10.1149/2.0851503jes.

[48] S. Walus , C. Barchasz , J. F. Colin , et al., “New Insight into the Working Mechanism of Lithium–Sulfur Batteries: In Situ and Operando X‐Ray Diffraction Characterization,” Chemical Communications 49 (2013): 7899, 10.1039/c3cc43766c. 23873017

[49] O. Pecher , J. Carretero‐Gonzalez , K. J. Griffith , and C. P. Grey , “Materials' Methods: NMR in Battery Research,” Chemistry of Materials 29 (2017): 213–242.

[50] O. Pecher , P. M. Bayley , H. Liu , Z. Liu , N. M. Trease , and C. P. Grey , “Automatic Tuning Matching Cycler (ATMC) in Situ NMR Spectroscopy as a Novel Approach for Real‐Time Investigations of Li‐and Na‐Ion Batteries,” Journal of Magnetic Resonance 265 (2016): 200–209, 10.1016/j.jmr.2016.02.008. 26938943

[51] Y. Kosugi , “Ammonium Sulfate as a Standard for ^33^S‐NMR Spectra,” Japan Oil Chemists' Society (YUKAGAKU) 42 (1993): 612–618, 10.5650/jos1956.42.612.

[52] L. A. Huff , J. L. Rapp , J. A. Baughman , P. L. Rinaldi , and A. A. Gewirth , “Identification of Lithium–Sulfur Battery Discharge Products Through ^6^Li and ^33^S Solid‐State MAS and ^7^Li Solution NMR Spectroscopy,” Surface Science 631 (2015): 295–300, 10.1016/j.susc.2014.07.027.

[53] Q. He , Y. Gorlin , M. U. M. Patel , H. A. Gasteiger , and Y.‐C. Lu , “Unraveling the Correlation Between Solvent Properties and Sulfur Redox Behavior in Lithium‐Sulfur Batteries,” Journal of the Electrochemical Society 165 (2018): A4027–A4033, 10.1149/2.0991816jes.

[54] A. Andersen , N. N. Rajput , K. S. Han , et al., “Structure and Dynamics of Polysulfide Clusters in a Nonaqueous Solvent Mixture of 1,3‐Dioxolane and 1,2‐Dimethoxyethane,” Chemistry of Materials 31 (2019): 2308–2319, 10.1021/acs.chemmater.8b03944.

[55] A. Dorai , J. Kawamura , and T. Omata , “Visualization of Polysulfide Dissolution in Lithium‐Sulfur Batteries Using In‐Situ NMR Microimaging,” Electrochemistry Communications 141 (2022): 107360, 10.1016/j.elecom.2022.107360.

