# Supplementary material for: Monitoring Redox Pathways and Performance Limitations in Lithium‐Sulfur Batteries Using In Situ 7/6Li and 33S NMR Spectroscopies
Source: Angew Chem Int Ed Engl. 2026 Apr 22;65(24):e25050. doi: 10.1002/anie.202525050 (PMC13245595; doi:10.1002/anie.202525050)
Supplement: Supplementary file 1 — The authors have cited additional references within the Supporting Information [6, 7, 19, 23, 33, 34, 38, 47, 49, 50, 51, 52, 53, 54, 55]. [file ANIE-65-e25050-s001.docx]

Supporting Information
©Wiley-VCH 2021
69451 Weinheim, Germany

Monitoring Redox Pathways and Performance Limitations in Lithium-Sulphur Batteries Using a Combination of *In Situ* ^7/6^Li and ^33^S NMR Spectroscopies

Jana B. Fritzke, Sunita Dey, Christopher A. O’Keefe, Jeongjae Lee, Kazuhiro Kamiguchi, Daisuke Mori, Yuri Nakayama and Clare P. Grey(s)*

**Abstract:** Lithium-sulphur (Li-S) batteries offer high capacity and reduced costs in comparison to the traditional lithium ion-systems. However, the complex series of redox mechanisms and structural transformations are often associated with different routes for cell failure. Therefore, a fundamental understanding of the underlying mechanism is essential to accelerate the development of these batteries. A combination of *operando* ^6/7^Li and ^33^S NMR spectroscopy is reported for the first time, providing real-time structural information on the reaction pathways of the sulphur redox processes. The evolution of the polysulphides (poly-S) in the electrolyte and dendrite formation on the anode were monitored with ^7^Li and ^6^Li NMR spectroscopy. Via ^33^S NMR experiments the exact onset of Li_2_S formation was determined, i.e., the point in the S-redox reaction where insoluble products form. On the basis of the correlation of the evolution of poly-S and Li_2_S, we can detect the entire redox pathway and identify performance limiting mechanisms. The accumulation of soluble poly-S resulting from an incomplete poly-S - S_8_ reaction during charge was identified as one process leading to capacity fade, while degradation via a poly-S shuttle mechanism was negligible, at least during the first few cycles.

DOI: 10.1002/anie.2021XXXXX


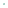


Table of Contents

[Experimental Procedures 2](#_Toc226823739)

[Cell assembly 2](#_Toc226823740)

[Galvanostatic cycling 3](#_Toc226823741)

[*Operando* NMR Spectroscopy 3](#_Toc226823742)

[Scanning Electron Microscopy (SEM) 3](#_Toc226823743)

[*Ex situ* Electron Paramagnetic Resonance Spectroscopy (EPR) 4](#_Toc226823744)

[Results and Discussion 4](#_Toc226823745)

[Electrochemical reaction in Li-S Batteries 4](#_Toc226823746)

[SEM images of the Li anode 4](#_Toc226823747)

[Static NMR measurement of Li_2_S 5](#_Toc226823748)

[^7^Li NMR Spectroscopy – Bulk magnetic susceptibility effect 6](#_Toc226823749)

[^7^Li NMR Spectroscopy – Diamagnetic peak 6](#_Toc226823750)

[^7^Li NMR Spectroscopy – *T_1_* measurements 7](#_Toc226823751)

[^7^Li NMR Spectroscopy with ^6^Li Anode 8](#_Toc226823752)

[Static ^33^S NMR -Cell components 9](#_Toc226823753)

[Static ^33^S NMR -Analysis of Li_2_S accumulation 10](#_Toc226823754)

[References 10](#_Toc226823755)

[Author Contributions 10](#_Toc226823756)

Experimental Procedures

Cell assembly

Cell assembly and air-sensitive material handling were done in an argon glovebox (MBraun, O_2_, H_2_O < 1 ppm). A capsule cell (NMR Service, eProbe) made out of PEEK (polyether ether ketone) was used for all *in situ* NMR experiments and has been described before.^[1]^ The cell and the battery stacking are shown in Figure S1. Lithium metal disks (15.6 mm diameter, 0.25 mm thick) as anode were purchased from PI-KEM, opened, and stored in an argon glovebox, used as received. The separator are two sheets of ADVANTEC GC-50 separators (dried at 100 °C under vacuum overnight). These separator sheets are soaked with 200 µL electrolyte. The electrolyte was prepared using 1 M lithium bis(trifluoromethane sulphonyl)imide (LiTFSI, Sigma-Aldrich, 99.95%) and 0.25 M lithium nitrate (LiNO_3_, Alfa Aesar, 99.999%) in 1,3-dioxolane (Acros Organics, anhydrous, 99.8%) and 1,2-dimethoxyethane (Merck, anhydrous, 99.9%) (DOL:DME in 1:1 volume ratio). The salts were dried for 20 h at 120 °C under a vacuum before use. The cathode is a carbon sulphur composite material containing 10wt% ^33^S or 50wt% S. Sulphur cathodes were prepared by mixing sulphur (99.999%, Wako) with Ketjen black carbon (Lion, EC600JD), styrene-butadine rubber (TRDA104, JSR) and carboxymethyl cellulose (BSH-6, DKS Co. Ltd.) binders. Enriched ^33^S (99%) was purchased from ISOFLEX USA. We have used the weight ratio of S/KB/SBR/CMC of 50/48/1.5/0.5 (sample 50wt% S) in most of the experiments. In ^33^S NMR experiments cathodes with the weight ratio S/KB/SBR/CMC of 10/88/1.5/0.5 (sample 10wt% S with 99% enriched ^33^S) is being used. Flat cathode sheets of 15 x 15 cm were made by pressing the mixed powder with a hand roller. Before assembling in capsule cells or coin cells, a small, fixed dimension of cathode sheet was mechanically pressed over stainless-steel mesh (Advent, 0.7 x 1.5 cm or 1 cm diameter) of same dimension using a regular hydraulic press (applied pressure 10 MPa).


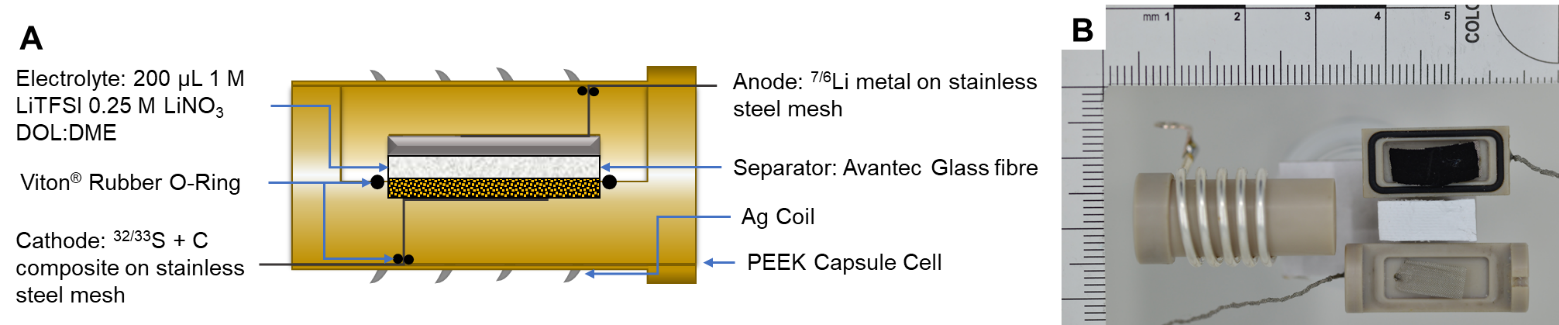


**Figure S1.** (A) Scheme of the Li-S cell stack inside of the capsule cell, showing the Li metal electrode, the carbon-sulphur cathode, the glass fibre separator soaked with the electrolyte and the stainless steel meshes as current collectors. (B) All components of an opened capsule cell in a coil with stainless steel mesh as current collector, the carbon-sulphur cathode and glass fibre separator, also showing the seals that help maintain the airtight cell and a cm scale-bar.

Galvanostatic cycling

BioLogic MPG2 cyclers were used for galvanostatic cycling of coin cells. A BioLogic VSP cycler was used during *operando* NMR experiments. EC-Lab software (V11.32) was used for data collection and processing. Galvanostatic cycling of the Li-S battery was done with C/10 for 10wt% S cathodes and C/20 for 50wt% S cathodes with a resting time of ca. 1 h.

*Operando* NMR Spectroscopy

The *operando* NMR experiments were conducted for ^7^Li NMR experiments on a Bruker Avance 300 MHz spectrometer (the Larmor frequency for ^7^Li being 116.6 MHz) and for the ^6^Li and ^33^S NMR experiments on a Bruker Avance III 700 MHz spectrometer (the Larmor frequency for ^6^Li being 103.0 MHz and for ^33^S 53.7 MHz) using a solenoidal Ag-coated Cu coil. The spectra were recorded using an *in situ* automatic-tuning-and-matching probe (ATM VT X *in situ* WB NMR probe, NMR Service, eProbe) that allows for an automatic recalibration of the NMR rf-circuit during an *in situ* electrochemistry experiment. The retuning of the rf-circuit becomes essential in order to quantify the NMR signals when the sample conditions are changing during the electrochemical cycling.^[2]^ The probe has highly shielded wire connections to the electrochemistry with low-pass filters (5 MHz) attached to the probe, minimizing the interferences between the NMR- and the electrochemistry circuit, as described in a previous publication.^[2]^ Overall, the *in situ* setup allows highly reproducible NMR measurements. All measurements had been conducted with the battery stacking oriented perpendicular to the static magnetic field B_0_.

For the ^7^Li NMR experiments single-pulses were used, with a recycle delay of 1 s and 128 scans for the metallic and 10 s and 16 transients recorded for the diamagnetic peak. This resulted in an experimental time of about 5 min in total. The shift of ^7^Li was referenced to 1 M LiCl in water at 0 ppm.

For the ^6^Li NMR experiments single-pulses were used, with a recycle delay of 10 s and 64 scans. This resulted in an experimental time of about 20 min. The shift of ^6^Li was referenced to 1 M LiCl in water at 0 ppm.

For the ^33^S NMR experiments Hahn echo-pulse were used, with a recycle delay of 1 s and 4096 scans. This resulted in an experimental time of about 20 min. The shift of ^33^S was referenced to a saturated solution of (NH_4_)_2_SO_4_ in water at -333 ppm that corresponds with CS_2_ at 0 ppm.^[3]^

The spectra were processed in the Bruker Topspin software using the automatic phase and baseline correction. Further data processing was done in R. The total intensity of the interested peaks was integrated over the defined ranges for ^7^Li -50-100 ppm and 310–220 ppm, for ^6^Li for -50-100 ppm and for ^33^S -400—200 ppm and normalised to the highest integral measured during the experiment.

Scanning Electron Microscopy (SEM)

After galvanostatic cycling with C/10, the coin cell was transferred into an Ar glovebox and disassembled after the 7^th^ charge. The Li metal electrode was mounted onto the SEM stage of the transfer module. (Kammrath & Weiss, type CT0) and dried under vacuum for 1 hour. The electrodes were not rinsed with a solvent before the measurement. The samples were transferred into the SEM chamber using the air sensitive transfer module under an inert atmosphere (Ar), without being exposed to air. SEM images were acquired with a Tescan CLARA 2 FEG-SEM instrument at an acceleration voltage of 3.0 kV.

*Ex situ* Electron Paramagnetic Resonance Spectroscopy (EPR)

*Ex situ* continuous wave EPR spectroscopy was carried out on an X-band benchtop Magnettech EPR spectrometer (E5000) that is set at a microwave frequency of 9.435 GHz. The electrolyte was extracted from cycled coin cells (C/10) in an Ar glovebox after the discharge to 1.8 V and charge to 2.6 V. The cycled electrolyte was transferred into quartz tubes and measured with a modulation field of 0.1 mT at a modulation frequency of 100 kHz. A microwave power of 50 mW was applied.

Results and Discussion

Electrochemical reaction in Li-S Batteries


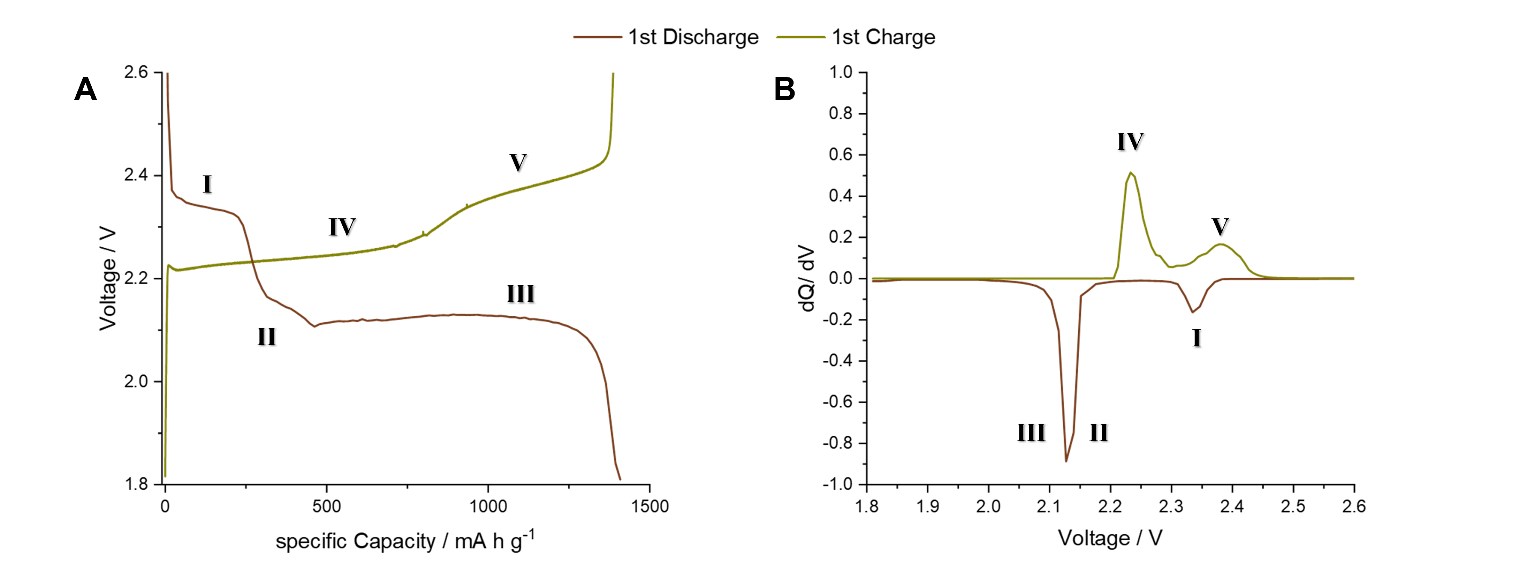


Figure S2. Voltage profile (A) and dQ/dV plot (B) of the 1^st^ cycle at C/20 of the *operando* Li-S battery.

Measuring a typical voltage profile (A) and corresponding dQ/dV plot (B) when cycling between 2.6 V and 1.8 V in a DOL:DME-based electrolyte are shown in Figure S2. The discharging process starts with a plateau (**I**) between 2.35 V and 2.30 V, that corresponds to a peak at 2.33 V in the dQ/dV plot. This plateau corresponds to the formation of long-chain poly-S. These long-chain poly-S are successively reduced to short-chain poly-S during the sloping region between 2.3 V and 2.1 V (**II**), which also contains a small flatter process just above 2.1 V. These characteristic features are represented as shoulder on the second peak in the dQ/dV plot at 2.14 V. The second and lower plateau (**III**) between 2.1 V and 2.05 V showing a peak at 2.12 V in the dQ/dV plot contributes to most of the capacity during discharge and corresponds to the reduction of poly-S.^[4,5]^

The charging voltage profile starts with a lower plateau (**IV**) between 2.24 V and 2.3 V and a peak at 2.23 V in the dQ/dV plot corresponding to the oxidation of insoluble sulphides to form dissolved poly-S on the cathode and Li metal plating on the anode. The large hysteresis in voltage between discharge and charge, is largely associated with the significant overpotential/activation energy associated with the oxidation of the insulating solid phase. A second plateau (**V**) between 2.3 V and 2.4 V with a corresponding peak in the dQ/dV plot at 2.38 V follows during which the poly-S are successively oxidized to elemental sulphur.

SEM images of the Li anode

The cycled cell was disassembled and the plated/charged anode was taken out for SEM investigation of the Li morphology. (Figure S3) The detected morphology of the cycled anode shows dendritic features with a diameter of less than 1 µm. This means, that the plated microstructure is smaller than the skin depth effect of the Li NMR experiments and the experiment is, therefore, fully quantitative.


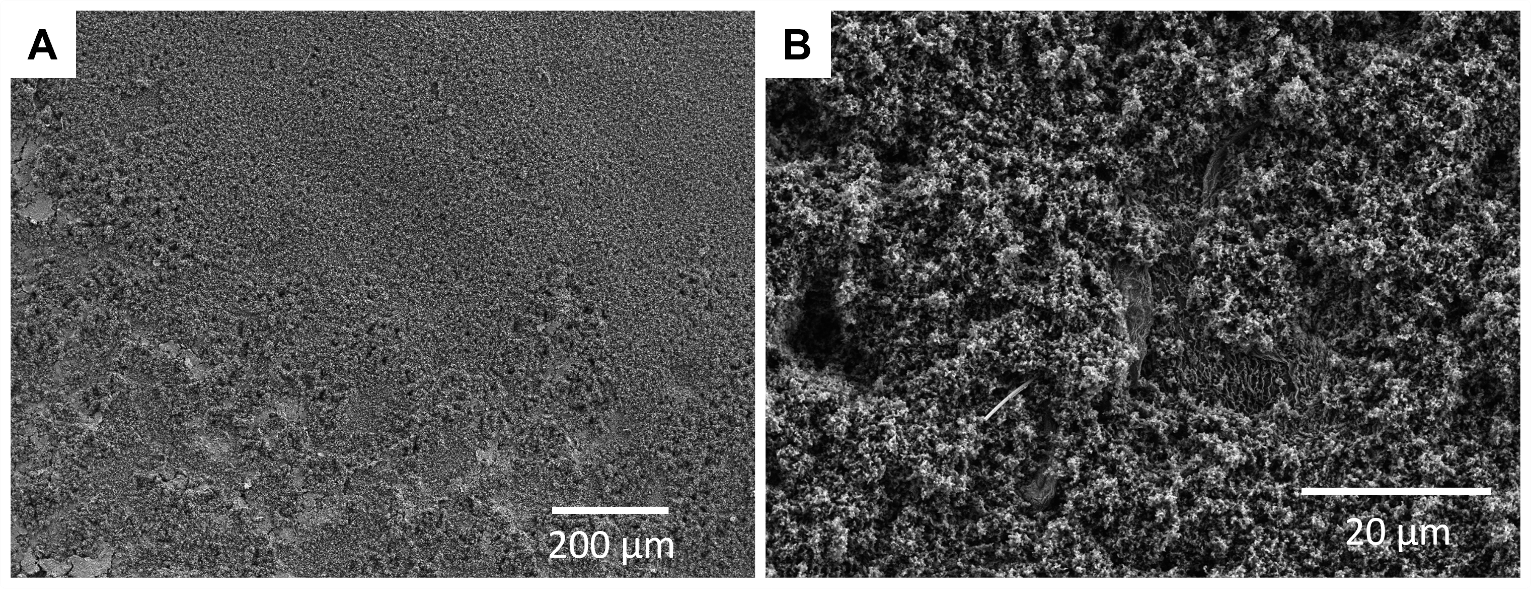
Figure S3. SEM images of the microstructures formed on Li metal anode after the 7^th^ charge cycling with C/10 at two different magnitudes.

Static NMR measurement of Li_2_S


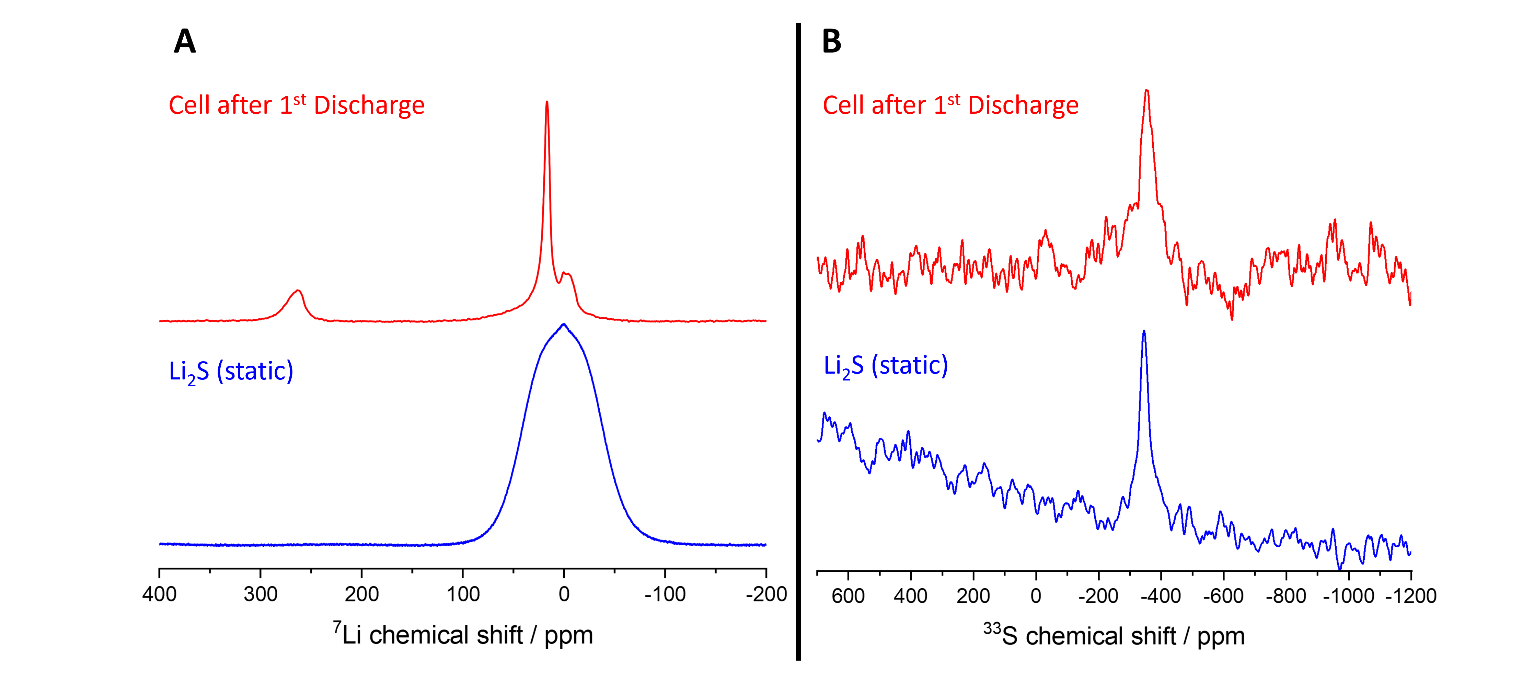


**Figure S4.** Static NMR measurements of Li_2_S (blue) in comparison to an *operando* cell after the 1^st^ Discharge (red): (A) ^7^Li NMR spectra and (B) ^33^S NMR spectra.

To obtain further insights into the detected environments of the ^7^Li and ^33^S nuclei, static NMR experiments of the reference sample Li_2_S was employed and compared with the spectra of a discharged cell. The ^7^Li NMR spectra of the cell shows multiple peaks in the diamagnetic region at 0 ppm that overlaps with the detected Li_2_S signal (0.28 ppm). (Figure S4 A) Measuring the Li_2_S reference in the static *in situ* probe shows a very broad line-shape, because the strong dipolar couplings, particularly between ^7^Li nuclei, are not averaged out by motion, and result in severely broadened NMR signals. Therefore, we can conclude that the broadening that was detected during the cycling of the *in situ* cell is largely arising from the formation of solid sulphide species e.g. Li_2_S.

In addition, ^33^S NMR spectrum was recorded of the Li_2_S reference, shown in Figure S4 B. The position of the signal detected in the *in situ* cell (-352.9 ppm) is in agreement with the signal of Li_2_S reference (-344.8 ppm). The ^33^S NMR peak of the cell is slightly broader and shifted up-field compared to the reference which is ascribed to BMS effects. ^[6]^

^7^Li NMR Spectroscopy – Bulk magnetic susceptibility effect

On placing a sample in a magnetic field (B_0_), a demagnetizing field is induced depending on the (i) macroscopic shape or the orientation of the object and (ii) the local heterogeneity and non-spherical shape of the crystallites relative to B_0_ This demagnetizing field adds to or opposes to B_0_ resulting in frequency shift and line broadening. The in situ measurements are done in static mode (magic angle spinning is absent), therefore the orientation of electrode films in in situ cells relative to the B_0_ will affect the shift and broadening. Moreover, the battery components, especially the conducting carbons and Li metal, which are the most significant contributors to the bulk magnetic susceptibility (BMS),^[7]^ result in complex shift and broadening patterns, which may evolve with cycling.

The Li metal resonance in the Li-S battery shows an orientation dependent shift of ca. 30 ppm while rotating the cell over 90°, as it was reported before in several publications.^[8,9]^ This shift change can be attributed to BMS effects caused by the temperature independent paramagnetism (TIP) of the metallic Li. This means the orientation dependence of the shift arises from the non-spherical shape of the Li metal, while the integral of the signal does not change.

**
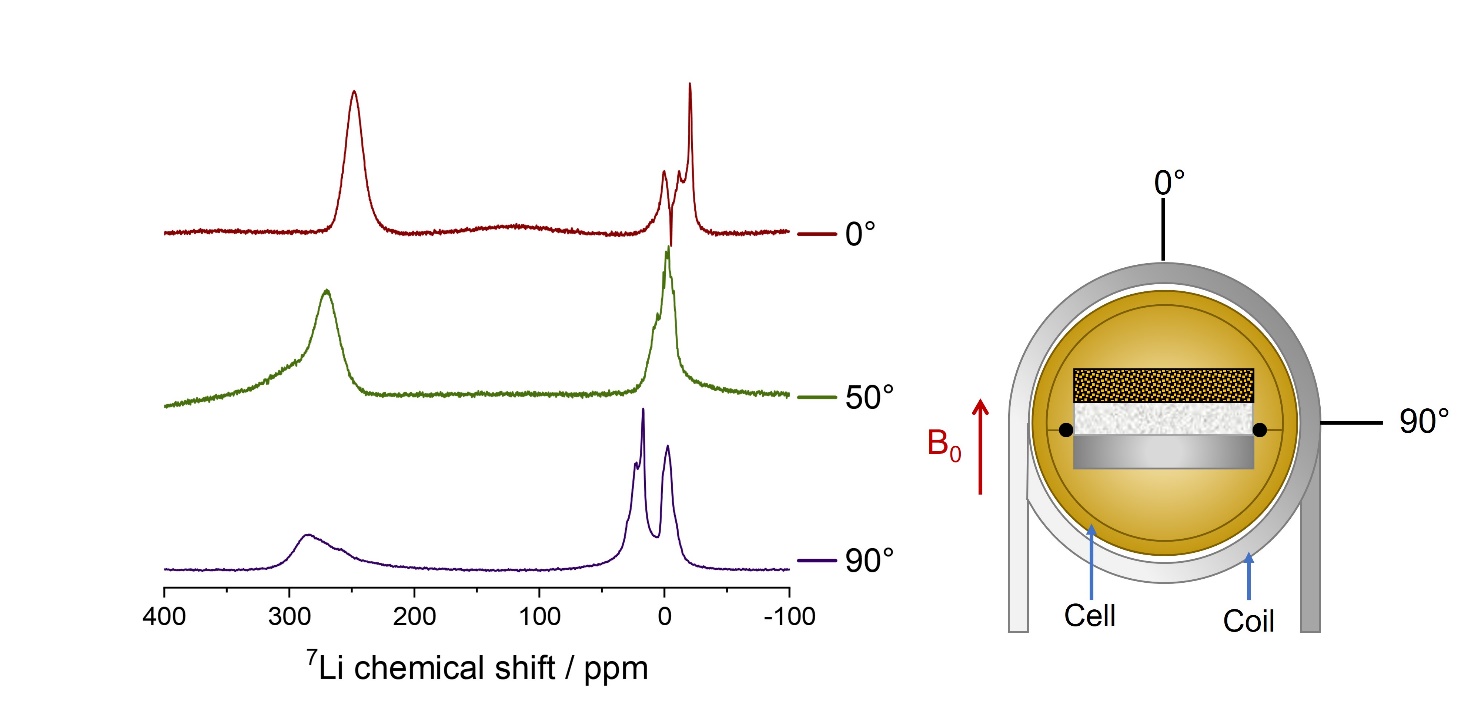
**

**Figure S5.** Bulk magnetic susceptibility effect on the ^7^Li shift of the detected peaks in the *in situ* Li-S cell. Spectra are given for diamagnetic and metallic Li with their short axes differently oriented with respect to B_0_ field. The scheme shows the orientation of the battery stack in the NMR coil at 90° (horizontal) with respect to B_0_.

In addition, there are noticeable shifts of the resonances seen in the diamagnetic region of the spectra (Figure S5). In the perpendicular position (90° towards B_0_) two distinct sharp signals are detectable at 20 ppm and -5 ppm that are overlapping with broader peaks. In comparison, measuring in parallel (0° towards B_0_) only one sharp signal at -18.9 ppm in addition to the broad features is detectable. Again, the difference in the position and broadening of the resonances are attributed to the magnetic susceptibility of the sample, due to the orientation of the electrodes. The multiple sharp features seen in the horizontal (90° to B_0_) orientation represent different species in different environments within the battery, which in principle could provide more information on spatial arrangements of the various components within the electrode.^[9]^ Therefore, this orientation was selected for all reported *operando* experiments. Bringing the cell close to the magic angle (50° towards B_0_) shows that the features merge to one sharp signal at ca. 0 ppm that proofs the BMS contribution to the chemical shifts.

The different BMS effect of the overlapping signals in the diamagnetic region can be, on one hand, associated with the carbon of the cathode. In previous studies it was shown that carbon is correlated with a large anisotropic diamagnetic susceptibility, due to the delocalised electrons. Species adsorbed on the surface or in the pores of the carbon are shifted to lower frequencies due to the interaction with the electron cloud, which is known as ring current effect.^[10]^ On the other hand, the electrolyte in the separator can also experience a susceptibility-induced shift, but it is much less significant than the shift seen in carbon materials.^[9]^

^7^Li NMR Spectroscopy – Diamagnetic peak

Measuring *operando* ^7^Li NMR spectroscopy of a Li-S battery shows a characteristic change of the peak shape and intensity during cycling. This is clearly visible when plotting the diamagnetic ^7^Li NMR spectra at different state of charge (SOC) (Figure S6 A) corresponding to the assigned regions in the voltage profile (Figure S6 B). It is clearly visible that the shape and intensity of the signal at 20 ppm is changing during cycling whereas the signal at -5 ppm remains unchanged. (Figure S6 B red) During the discharge plateau **I** and the sloping region **II**, the intensity of the signal at 20 ppm increases, due to the formation of soluble poly-S. In the following 2^nd^ discharge plateau **III**, the intensity decreases again, but this is accompanied with a broadening which can be explained by the formation of solid species. During the 1^st^ charge plateau **IV**, the intensity of the peak at 20 ppm increases again, because of the oxidation of Li_2_S forming soluble poly-S. In the 2^nd^ charge plateau **V**, the intensity and the broadening decreases, as the Li_2_S is consumed while diamagnetic Li^+^ is plated on the anode.

**
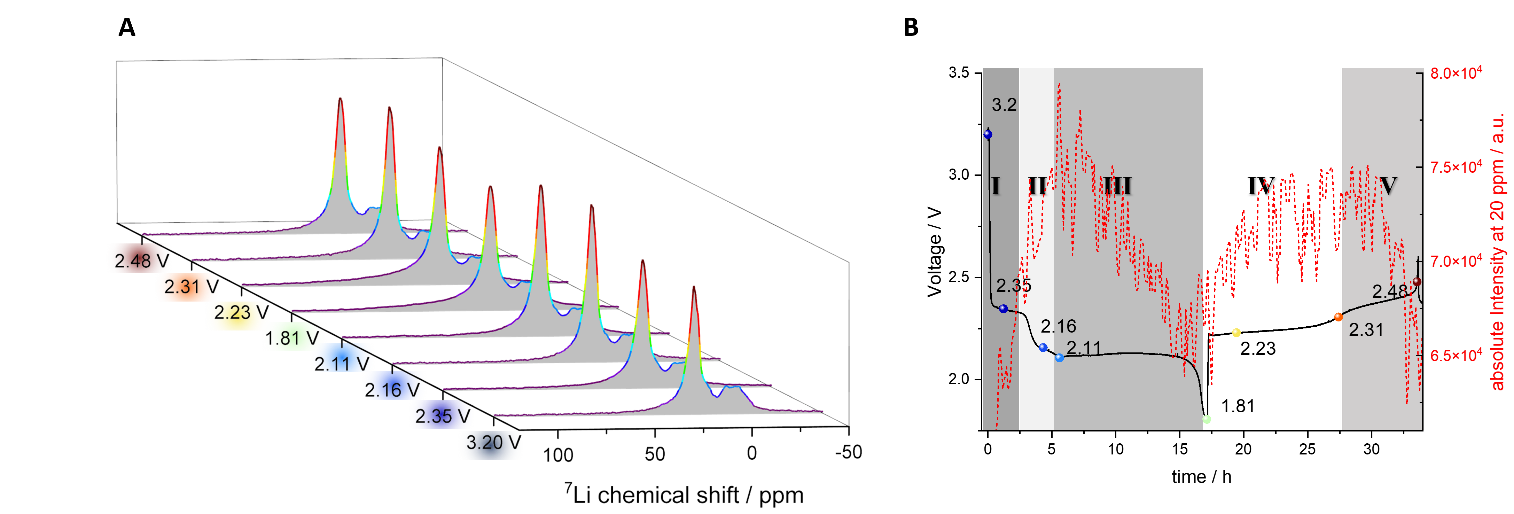
**

**Figure S6.** Selected ^7^Li NMR spectra (A) at defined states of charge during the first cycle of the operando experiment of a Li-S battery and voltage profile with the assigned regions in grey (**I-V**) and the absolute Intensities of the peak at 20 ppm in red (B).

^7^Li NMR Spectroscopy – *T_1_* measurements

The spin lattice relaxation times (*T_1_*) of various ^7^Li species were acquired *in situ*, at different states of charge, by applying a saturation recovery pulse sequence. Plotting the *T_1_* times vs. the depth of discharge (DOD) or state of charge (SOC) of the cell (Figure S7) shows firstly, very different *T_1_* times for the diamagnetic peaks at approximately 20 ppm (red dots) and at -5 ppm (green triangles) and the metal peak at 264 ppm (black squares). Secondly, *T_1_* times of the diamagnetic peaks depend to a degree on the state of charge and thus the redox reactions, whereas the *T_1_* times of the metal peak at 264 ppm (black squares) is both much shorter, and also independent of the redox processes. The former changes in *T_1_* are ascribed to the formation of the different electrolyte environments caused by the changes in formation of different poly-S clusters and charge compensating Li^+^ ions during cycling, and the accompanying changes in electrolyte viscosity. There is also more cell-to-cell variation in the *T_1_* times of the diamagnetic peaks, while the Li metal ones, not surprisingly, remain essentially constant, from cell to cell.

At the beginning of the cycling, similar *T_1_* times are seen for the diamagnetic signals (approximately 800 ms), but a significant drop in the *T_1_* time to 400 ms of the -5 ppm resonance is seen. The relaxation times then only drop very slightly for both peaks at the end of the 2.3 V discharge plateau (**I**). While the large variation in *T_1_* times seen between runs precludes a more in-depth analysis at this time, we note that *T_1_* times are likely influenced by two factors. The first is a change in viscosity as Li^+^ ions move closer to the carbon electrode to charge compensate the poly-S anions, and more poly-S anions are dissolved into solution. For example, the presence of long-chain, poly-S Li_2_S_n_ (n > 5), which exist as highly soluble (in the ether-based solvents) small cluster or monomers^[11]^, could result in significant changes in viscosity. The *T_1_* times will depend on the timescale of the rotational motions of the various complexes and the sizes of the resulting rapidly fluctuating local fields. The largest fluctuating local fields in the diamagnetic electrolytes are caused by changes in the Li quadrupolar coupling constants of the complexes, including Li^+^ ether complexes and any binding to any poly-S anions. Increases in viscosity should result in slower translational and rotational motion and thus in shorter *T_1_* times, although variable temperature NMR studies are needed to confirm this. The second factor is due to relaxation effects from paramagnetic ions. These include the possible formation of stable LiS_3_^•^ radicals as reported elsewhere^[12]^, and also radicals present on the carbon electrode surfaces. An *ex situ* EPR investigation of the cycled electrolyte shows a small concentration of stable radicals after discharge that are consumed during charge (Figure S7 B). The main conclusion that emerges from this *T_1_* study is that there are two sets of ^7^Li spins with very different local chemical environments – i.e., the electrolyte composition near the carbon is very different from that in the separator. It is also noteworthy that all the detected *T_1_* relaxation times are, however, much shorter than those of the pure crystalline Li_2_S material (131±12 s), as reported in the literature.^[13]^ This again confirms that the signals we are detecting are dominated by the shorter relaxing dissolved Li^+^ ions.


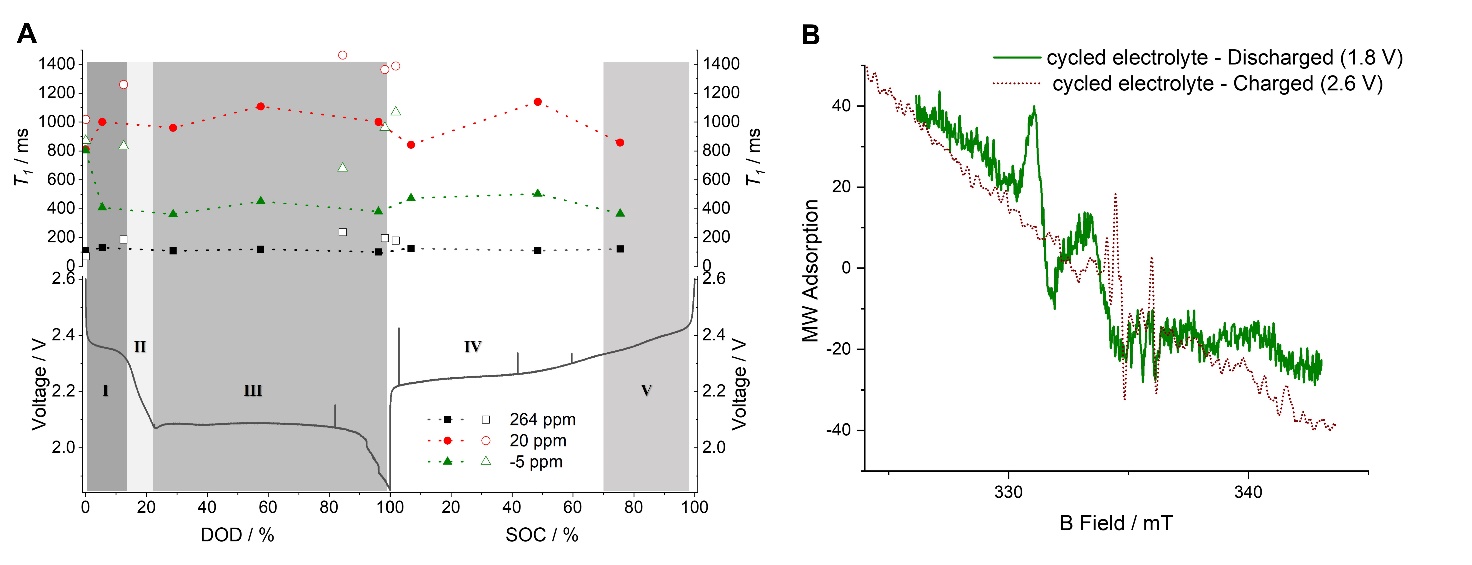


**Figure S7. A** *In situ* ^7^Li NMR spectroscopy *T*_1_ measurement of a Li-S battery at different states of charge using saturation recovery pulse sequences. The *T_1_* relaxation times of the paramagnetic peak at 264 ppm (black squares, filled) and diamagnetic peaks at 20 ppm (red dots, filled) and -3 ppm (green triangles, filled) and the repeat measurements in the unfilled marks plotted versus DOD/SOC. **B** *Ex situ* EPR measurements of the electrolyte after the discharge to 1.8 V (solid, green) and charge to 2.6 V (dotted, red) showing the reversible appearance of radicals during cycling.

^7^Li NMR Spectroscopy with ^6^Li Anode

Measuring *operando* ^7^Li NMR spectroscopy of a cell with an enriched ^6^Li metal anode is consistent with our hypothesis that there is the exchange between the ^6^Li metal and the ^7^Li species in the electrolyte. Quantifying the Li metal peak during cycling, shows a decrease of the integral during the 1^st^ discharging plateau (Figure S8 A). This can be explained by a stripping of ^7^Li from the surface of the anode that had been accumulated due to the fast exchange between the ^6^Li metal and the ^7^Li electrolyte. During the 2^nd^ discharge plateau, the integral of the metal stays constant due to further stripping of ^6^Li from the anode. From the beginning of the charging an increase of the integral is detectable, which can be explained by the formation of microstructured Li metal from a mixture of ^6^Li and ^7^Li in the electrolyte after the 1^st^ discharge. In the following cycles, the behaviour of the metallic peak is similar to that of reported *operando* ^7^Li NMR spectroscopic experiment, where the accumulation of microstructures is detected.

However, the diamagnetic region shows an increase of the integral during the first plateau and the sloping region (Figure S8 B). This indicates that more diamagnetic ^7^Li species are formed due to the stripping of metal during the redox reaction. This diamagnetic ^7^Li comes from the surface of the anode, that underwent an exchange of the ^6^Li metal and ^7^Li from the electrolyte.^[14]^ From the beginning of the 2^nd^ discharge plateau, the integral decreases, because of the formation of ^6^Li-based poly-S is superimposed with further exchange with the metal and the formation of a solid species, such as Li_2_S, which are present in the static measurement as very broad resonances.

On starting the charging process, the diamagnetic peak decreases further due to the plating and formation of Li metal instead. A slight drop at the end of the charging process is seen, when the Li_2_S is completely consumed and the soluble Li species are plated.


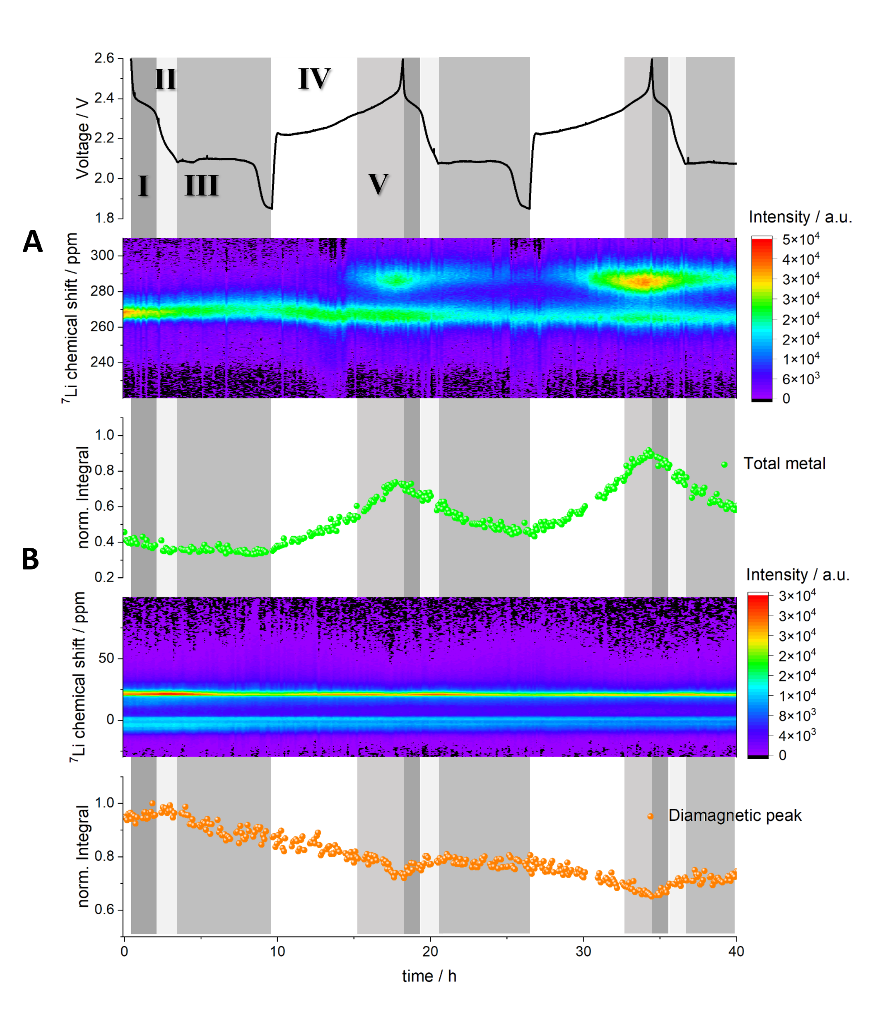


**Figure S8.** *Operando* ^7^Li NMR spectroscopy of a Li-S battery with a ^6^Li metal anode and corresponding voltage profile with a C-rate of C/10 with the different plateaus in grey (**I**-**V**). The characteristic (A) Signal (between 200 and 350 ppm) on the top and normalised integral of the peak corresponding to total metal (bottom). (B) Signal (between -50 and 100 ppm) (top) and normalised integrals of the diamagnetic peaks (bottom).

Static ^33^S NMR -Cell components

To get more information where the Li_2_S is formed inside of the cell, a cycled *in situ* cell was disassembled after the 2^nd^ discharge and a ^33^S NMR spectrum for every component was measured individually. (Figure S9) There is no Li_2_S is detected on the anode, which we can conclude that the formation of Li_2_S on the anode during SEI formation and poly-S shuttle is not as pronounced as it would lead to capacity fade. In comparison, on the separator a small Li_2_S peak is detected which can be explained by attached residues of the cathode material after disassembling the cell. The largest peak of Li_2_S is visible on the cathode material confirming that the conversion of soluble poly-S to Li_2_S happens as desired in the cathode.


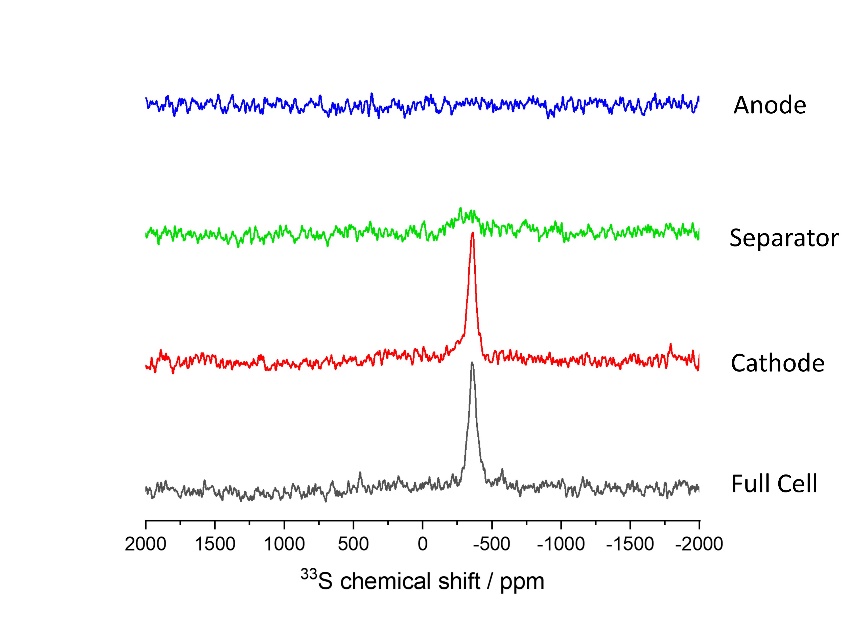


**Figure S9.** Comparison of the ^33^S NMR spectra of the full cell with the cell components of a Li-S Battery, which was disassembled after the 2^nd^ discharge: Anode (blue), Separator (green), Cathode (red) and assembled full cell (grey) from the top.

Static ^33^S NMR -Analysis of Li_2_S accumulation

Li_2_S accumulation has frequently been proposed as a primary degradation mechanism in Li–S batteries; therefore, quantifying both its formation and reversibility during cycling is critical for assessing its contribution to capacity fade.

The cell initially contains approximately 1 mg of ^33^S. In the first discharge, ~70% of the theoretical capacity is accessed, with ~50% arising from the second discharge plateau (plateau III), corresponding to the onset of Li_2_S formation. Accordingly, a full discharge is expected to form ~0.6 mg of Li_2_S in the cell.

In the fully discharged state of the first cycle, a signal intensity of 9.8∙10^6^ is observed, corresponding to a signal-to-noise ratio (SNR) of ~14 (Figure S10A). Using a SNR of 2 (commonly defining the limit of detection) the minimum detectable amount of Li_2_S is ~0.08 mg. After the subsequent charge, the Li_2_S signal falls below this detection limit, indicating that more than 87% of the Li_2_S formed during discharge is removed in the first cycle.

If the measured capacity fade were entirely due to irreversible Li_2_S accumulation, then after the third charge (remaining capacity ~54%) approximately 0.23 mg of Li_2_S would be expected to persist in the cell. Experimentally, after the third discharge, a signal intensity of ~1.2∙10^7^ is observed, whereas after charge a residual signal with an SNR of 2.69 remains (Figure S10A), corresponding to ~0.11 mg of Li_2_S. This value is substantially lower than that predicted from capacity loss alone, demonstrating that Li_2_S accumulation cannot fully account for the degradation. The residual Li_2_S may reside on either the cathode or the anode.


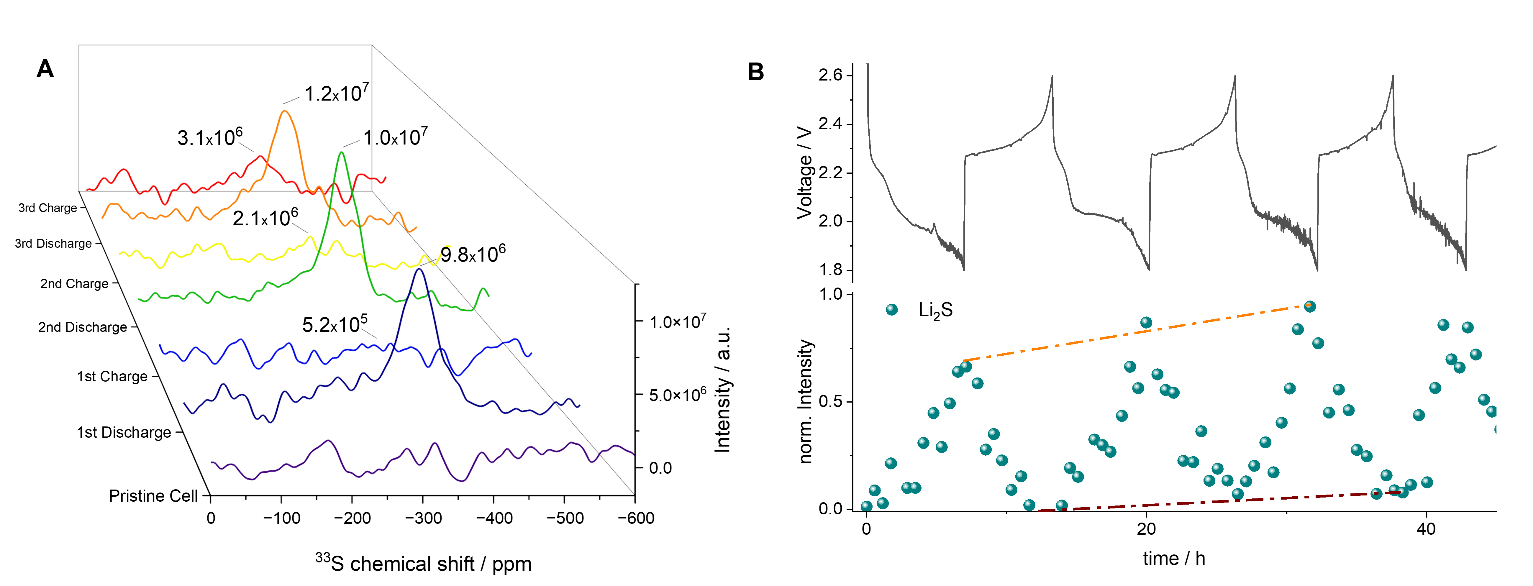


**Figure S10. A** Plotted spectra of the discharged and charged cell during 3 cycles. Three spectra, obtained by signal averaging over 20 minutes, are added together for each spectrum to increase the SNR by a factor of √3 and signals are denoted with intensities showing the increase of Li_2_S during cycling. **B** Voltage profile with a C-rate of C/10 (top) and normalised integral of the Li_2_S signal (blue dots) with the slope illustrating the change in Li_2_S intensity at the top of charge (brown line) and bottom of discharge (orange line).

A complementary perspective is obtained by tracking the maximum Li_2_S signal at the top of charge (Figure S10B, brown line) and at the bottom of discharge (Figure S10B, orange line). The slopes describing the evolution of Li_2_S intensity with cycle number are similar within the SNR limit, indicating only minor net accumulation on the cathode. While the Li_2_S signal increases slightly more during discharge than during charge (by ~20% per cycle), most Li_2_S formed during discharge is removed on charging. Any Li_2_S accumulation during charge is likely dominated by deposition on the anode, associated with poly-S shuttling during Li plating, but does not exceed ~15% per cycle after the first cycle. However, we note that no Li_2_S signal is seen in the ^33^S spectrum of the anode after the second discharge – taken of the disassembled cell - suggesting that Li_2_S accumulation is minimal in the 1st 1.5 cycles but that the subsequent Li_2_S accumulation likely occurs on both electrodes.

Taken together, these analyses show that the detection limit is sufficiently low to quantitatively resolve inactive Li_2_S at levels capable of explaining the observed capacity fade. The absence of such accumulation in the early cycles therefore supports the conclusion that Li_2_S accumulation is not the dominant degradation mechanism during the initial stages of cycling.

# References

[1] O. Pecher, J. Carretero-Gonzalez, K. J. Griffith, C. P. Grey, “Materials’ methods: NMR in battery research” *Chemistry of Materials* **2017**, *29*, 213–242.

[2] O. Pecher, P. M. Bayley, H. Liu, Z. Liu, N. M. Trease, C. P. Grey, “Automatic Tuning Matching Cycler (ATMC) in situ NMR spectroscopy as a novel approach for real-time investigations of Li-and Na-ion batteries” *Journal of Magnetic Resonance* **2016**, *265*, 200–209.

[3] Y. Kosugi, “Ammonium Sulfate as a Standard for 33S-NMR Spectra” *J. Jpn. Oil Chem. Soc. (YUKAGAKU)* **1993**, *42*, 612–618.

[4] Y. Luo, Z. Fang, S. Duan, H. Wu, H. Liu, Y. Zhao, K. Wang, Q. Li, S. Fan, Z. Zheng, W. Duan, Y. Zhang, J. Wang, “Direct Monitoring of Li_2_S_2_ Evolution and Its Influence on the Reversible Capacities of Lithium‐Sulfur Batteries” *Angew. Chem. Int. Ed.* **2023**, *62*, e202215802.

[5] C. Barchasz, F. Molton, C. Duboc, J. C. Leprêtre, S. Patoux, F. Alloin, “Lithium/sulfur cell discharge mechanism: An original approach for intermediate species identification” *Anal. Chem.* **2012**, *84*, 3973–3980.

[6] L. A. Huff, J. L. Rapp, J. A. Baughman, P. L. Rinaldi, A. A. Gewirth, “Identification of lithium-sulfur battery discharge products through 6Li and 33S solid-state MAS and 7Li solution NMR spectroscopy” *Surf. Sci.* **2015**, *631*, 295–300.

[7] L. Zhou, M. Leskes, A. J. Ilott, N. M. Trease, C. P. Grey, “Paramagnetic electrodes and bulk magnetic susceptibility effects in the in situ NMR studies of batteries: Application to Li1.08Mn 1.92O4 spinels” *Journal of Magnetic Resonance* **2013**, *234*, 44–57.

[8] R. Bhattacharyya, B. Key, H. Chen, A. S. Best, A. F. Hollenkamp, C. P. Grey, “In situ NMR observation of the formation of metallic lithium microstructures in lithium batteries” *Nat. Mater.* **2010**, *9*, 504–510.

[9] N. M. Trease, L. Zhou, H. J. Chang, B. Y. Zhu, C. P. Grey, “In situ NMR of lithium ion batteries: Bulk susceptibility effects and practical considerations” *Solid State Nucl. Magn. Reson.* **2012**, *42*, 62–70.

[10] R. K. Harris, T. V Thompson, P. R. Norman, C. Pottage, *Phosphorus-31 NMR studies of adsorption onto activated carbon*, **1999**.

[11] A. Andersen, N. N. Rajput, K. S. Han, H. Pan, N. Govind, K. A. Persson, K. T. Mueller, V. Murugesan, “Structure and Dynamics of Polysulfide Clusters in a Nonaqueous Solvent Mixture of 1,3-Dioxolane and 1,2-Dimethoxyethane” *Chemistry of Materials* **2019**, *31*, 2308–2319.

[12] Q. Wang, J. Zheng, E. Walter, H. Pan, D. Lv, P. Zuo, H. Chen, Z. D. Deng, B. Y. Liaw, X. Yu, X. Yang, J.-G. Zhang, J. Liu, J. Xiao, “Direct Observation of Sulfur Radicals as Reaction Media in Lithium Sulfur Batteries” *J. Electrochem. Soc.* **2015**, *162*, A474–A478.

[13] M. U. M. Patel, I. Arčon, G. Aquilanti, L. Stievano, G. Mali, R. Dominko, “X-ray absorption near-edge structure and nuclear magnetic resonance study of the lithium-sulfur battery and its components” *ChemPhysChem* **2014**, *15*, 894–904.

[14] A. B. Gunnarsdóttir, S. Vema, S. Menkin, L. E. Marbella, C. P. Grey, “Investigating the effect of a fluoroethylene carbonate additive on lithium deposition and the solid electrolyte interphase in lithium metal batteries using: In situ NMR spectroscopy” *J. Mater. Chem. A Mater.* **2020**, *8*, 14975–14992.

# Author Contributions

JBF performed the *operando* NMR measurements on LiS batteries, including cell assembly, NMR optimization and data processing. SD and CAO prepared electrochemical cells and assisted with NMR measurements. JBF and SD processed the experimental data, performed the analysis, drafted the manuscript and designed the figures. KK, DM and YN developed and prepared the cathode material for the study. JBF, SD, CAO, JL and CPG contributed to the interpretation of the results. CPG and YN designed and directed the project. All authors provided critical feedback and helped shape the research, analysis, and manuscript.
